# A New Combination of Radio-Frequency Coil Configurations Using High-Permittivity Materials and Inductively Coupled Structures for Ultrahigh-Field Magnetic Resonance Imaging

**DOI:** 10.3390/s22228968

**Published:** 2022-11-19

**Authors:** Jeung-Hoon Seo, Young-Seung Jo, Chang-Hyun Oh, Jun-Young Chung

**Affiliations:** 1 Neuroscience Research Institute, Gachon University, Incheon 21988, Republic of Korea; 2 Department of Electronics and Information Engineering, Korea University, Sejong 30019, Republic of Korea; 3 Department of Neuroscience, College of Medicine, Gachon University, Incheon 21565, Republic of Korea

**Keywords:** inductively coupled wireless structure, high-permittivity material, finite-difference time domain method, multichannel RF coil, birdcage RF coil, 7.0 T MRI

## Abstract

In ultrahigh-field (UHF) magnetic resonance imaging (MRI) system, the RF power required to excite the nuclei of the target object increases. As the strength of the main magnetic field ($B_{0}$ field) increases, the improvement of the RF transmit field ($B_{1}^{+}$ field) efficiency and receive field ($B_{1}^{-}$ field) sensitivity of radio-frequency (RF) coils is essential to reduce their specific absorption rate and power deposition in UHF MRI. To address these problems, we previously proposed a method to simultaneously improve the $B_{1}^{+}$ field efficiency and $B_{1}^{-}$ field sensitivity of 16-leg bandpass birdcage RF coils (BP-BC RF coils) by combining a multichannel wireless RF element (MCWE) and segmented cylindrical high-permittivity material (*sc*HPM) comprising 16 elements in 7.0 T MRI. In this work, we further improved the performance of transmit/receive RF coils. A new combination of RF coil with wireless element and HPM was proposed by comparing the BP-BC RF coil with the MCWE and the *sc*HPM proposed in the previous study and the multichannel RF coils with a birdcage RF coil-type wireless element (BCWE) and the *sc*HPM proposed in this study. The proposed 16-ch RF coils with the BCWE and *sc*HPM provided excellent $B_{1}^{+}$ field efficiency and $B_{1}^{-}$ field sensitivity improvement.

## 1. Introduction

Advances in noninvasive 3-dimensional (3D) medical imaging such as ultrasound, computed tomography, and magnetic resonance imaging (MRI) have contributed to the early diagnosis of neurodegenerative diseases with enhanced accuracy. Among them, MRI provides a high signal-to-noise ratio (SNR) and contrast-to-noise ratio (CNR), as well as exquisite spatial resolution. In addition, MRI has been highly utilized in clinical practice, as it can provide both anatomical and functional images.

However, over the past 20 years, the main magnetic field ($B_{0}$ field) strength of MRI has remained limited to 3.0 T [1,2,3,4], and the clinical use of ultrahigh-field (UHF) MRI ($B_{0}$ field: 7.0 T or higher) has been delayed despite its advantages, such as high SNR, CNR, and spatial resolution, owing to the increased $B_{0}$ field strength.

The recent approval of 7.0 T MRI scanners by the United States Food and Drug Administration (FDA) can greatly contribute to the diagnosis of neurodegenerative diseases, such as Alzheimer’s disease [5,6,7,8,9,10]. In particular, 7.0 T MRI can help detect and distinguish subtle and potentially treatable lesions that cannot be detected or optimally evaluated at 3.0 T [11,12,13,14,15,16,17].

Nevertheless, 7.0 T MRI could not be widely used in clinical applications due to radio-frequency (RF) safety issues because RF coils require relatively high RF power higher than 3.0 T [18,19,20,21,22,23,24,25]. For instance, the use of the 7.0 T MRI system approved by the U.S. FDA is subject to clinical permission to acquire only head and extremities (arms and legs) images. The 7.0 T MRI system requires a large amount of RF energy for whole-body imaging, causing tissue heating upon RF exposure [26,27,28,29,30,31,32] and increasing the specific absorption rate (SAR) depending on the electric field (|E| field) concentration [33,34,35,36,37,38,39,40,41,42].

The improvement of RF coil performance plays an essential role in overcoming the RF safety issues related to UHF MRI, which requires the acquisition of images using less RF power to secure RF safety as the $B_{0}$ field strength increases. For this reason, RF field ($B_{1}$ field) sensitivity and uniformity of the RF coil have become important design elements [43,44,45,46,47]. In the MRI system, the RF power required to excite the nuclei of the target object increases with the $B_{0}$ field strength. As the $B_{0}$ field intensity increases, the B_1_ field sensitivity of the RF coil used in UHF MRI becomes particularly important because greater RF power is required to excite the proton (^1^H) nuclei of the human body using RF pulses [48,49,50,51,52,53,54,55,56,57,58].

To improve $B_{1}$ field efficiency, the simultaneous improvement of the RF transmit field ($B_{1}^{+}$ field) efficiency and receive field ($B_{1}^{-}$ field) sensitivity of the RF coil is required [59,60,61,62,63,64]. Previous studies have been conducted to improve the $B_{1}^{+}$ field efficiency and the $B_{1}^{-}$ field sensitivity separately; however, UHF MRI requires the improvement of both the $B_{1}^{+}$ field efficiency [47,65,66,67,68,69] and the $B_{1}^{-}$ field sensitivity [47,70,71,72,73,74,75,76] simultaneously.

Therefore, a method for simultaneously improving the $B_{1}^{+}$ field efficiency and the $B_{1}^{-}$ field sensitivity was previously proposed to obtain a UHF MR image with a minimum SAR. The proposed RF coil configuration was based on the combination of high-permittivity materials and inductively coupled elements to improve the $B_{1}^{+}$ field efficiency and the $B_{1}^{-}$ field sensitivity, determined by electromagnetic field (EM field) simulations [47].

To explain the RF coil configurations proposed in our previous work more specifically, the EM field simulation was performed using a bandpass-type birdcage (BP-BC) RF coil for RF transmission and reception because BP-BC RF coils provide the most uniform and the highest $B_{1}$ field efficiency in UHF MRI [77,78,79,80]. Additional structures were proposed using segmented cylindrical high-permittivity material (*sc*HPM) to improve $B_{1}^{+}$ field efficiency and a multichannel wireless element (MCWE) to improve $B_{1}^{-}$ field sensitivity. The suitability of their use in 7.0 T MRI has been confirmed in a previous study, but further investigation is required to obtain their combination to further improve the $B_{1}^{+}$ field efficiency and the $B_{1}^{-}$ field sensitivity.

First, the disadvantage of the BP-BC RF coil combinations with *sc*HPM and MCWE is that unwanted frequencies can be applied to *sc*HPM by the extremely narrow mode space of the BP-BC RF coil in UHF MRI. The $B_{1}^{+}$ field generated around each leg of BC RF coil is drawn according to Maxwell’s right-hand rule. Each leg of the BC RF coil is driven by a sinusoidal current, but the peak current of each successive leg is delayed by 360°/16 (number of legs) = 22.5°. This is a homogeneous mode of the BC RF coil. In the resonance spectrum (in S-parameters) of the BC RF coil, the BC RF coil has various modes equal to half the number of legs of the BC RF coil, except for the end-ring modes. However, the modes of these BC RF coils have an extremely narrow mode space in the UHF MRI. For this reason, a decrease in $B_{1}^{+}$ field efficiency was expected; thus, the BP-BC RF coil used as the transmit/receive (Tx/Rx) RF coil was replaced with the 16 channel (16-ch) RF coil. We also improved the $B_{1}^{-}$ field sensitivity of the 16-element MCWE using large-volume BC RF coils as the wireless element (WE) owing to an increase in the inductively coupled area. The dimensions of the MCWE and *sc*HPM were defined as the size between the legs of the BP-BC RF coil. The $B_{1}$ field distribution of the BP-BC RF coil is generated in the vertical direction of the closed loop between the legs. If the MCWE and *sc*HPM dimensions exceed the size between the legs of the BP-BC RF coil, the RF wave may be distorted horizontally, and signal sensitivity may be reduced, so the dimensions and number of MCWE and *sc*HPM were set to a size that can minimize RF wave distortion.

Therefore, in this study, we propose a new combination of RF coil configuration that provides superior $B_{1}^{+}$ field efficiency and $B_{1}^{-}$ field sensitivity at 7.0 T MRI compared to the previously proposed combinations of the *sc*HPM and the MCWE. To this end, EM field simulations were performed by alternating the role of the BP-BC RF coil used as a Tx/Rx RF coil and the MCWE used as a WE to improve $B_{1}^{-}$ field sensitivity, except for *sc*HPM (which has already been verified to improve $B_{1}^{+}$ field efficiency).

Moreover, we compared the 16-ch RF coil with or without the BCWE (*w/wo*-BCWE) and the BP-BC RF coil with or without the MCWE (*w/wo*-MCWE) using EM field simulations. Thus, the optimal configuration of the BP-BC RF coil with MCWE and *sc*HPM combinations (BP-BC RF coil + *sc*HPM – *w/wo*-MCWE) and the 16-ch RF coil with BCWE and *sc*HPM combinations (16-ch RF coil + *sc*HPM – *w/wo*-BCWE) was identified with enhanced $B_{1}^{+}$ field efficiency and $B_{1}^{-}$ field sensitivity. Through the alternating use of the Tx/Rx RF coil and the WE, it was possible to determine which cases provided further improved $B_{1}^{+}$ field efficiency and $B_{1}^{-}$ field sensitivity. The performance of each RF coil combination was compared using EM field analysis under unnormalized (|$B_{1}^{+}$|, |$B_{1}^{-}$|, and |E| fields) and normalized (|$B_{1}^{+}$| field and SAR) conditions. The EM field simulations confirmed that the advanced form of the 16-ch RF coil + *sc*HPM – *w/wo*-BCWE configuration provided enhanced $B_{1}^{+}$ field efficiency and $B_{1}^{-}$ field sensitivity compared to the BP-BC RF coil + *sc*HPM – *w/wo*-MCWE at 7.0 T.

## 2. Materials and Methods

### 2.1. EM Field Simulation Setup

To evaluate the performance of the BP-BC RF coil + *sc*HPM – *w/wo*-MCWE configuration and the 16-ch RF coil + *sc*HPM – *w/wo*-BCWE configuration, 3D modeling and EM field calculations were performed using the FDTD method based on xFDTD simulation software version 6.6 (Remcom, Inc., State College, PA, USA) [81].

The components of the EM field simulation model were divided into three types: the Tx/Rx RF coil (BP-BC RF coil and the 16-ch RF coil) for RF transmission and reception, the WE (MCWE and BCWE) for $B_{1}^{-}$ field sensitivity improvement, and the *sc*HPM for $B_{1}^{+}$ field efficiency improvement.

As Tx/Rx RF coils for human head MR imaging, the BP-BC RF coil and the 16-ch RF coil were designed to have the same diameter (330 mm) and length (150 mm). The BP-BC RF coil consists of 16 legs with a BP-BC type structural design, and the 16-ch RF coil consists of 16 surface elements. The BP-BC RF coil and the 16-ch RF coil were operated in the Tx/Rx mode, and they provided uniform RF distribution to the conductor using current sources (1 A) with a sinusoidal RF pulse. Current RF sources are used assuming an ideal current distribution, and RF coupling between RF elements is neglected [82,83,84,85,86,87,88,89,90]. The BP-BC RF coil consists of 16 legs with 80 current RF sources, and the 16-ch RF coil consists of 128 current RF sources (8 current RF sources per single channel). For ideal current driving condition through the micro-strip line of the transmission RF coil, multiple current RF sources were applied instead of the capacitors used for tuning and matching the RF coil. In addition, the micro-stripe lines constituting the RF coil were composed of a perfect electric conductor (PEC) instead of copper. Where the current intensity and geometrical phase of RF coils are defined at each current driving point, the EM field distribution can be calculated in an actual MRI experiment assuming target resonance RF frequency tuning and impedance matching with a tuning and matching capacitor. For $B_{1}^{-}$ field sensitivity improvement, the WE was designed and compared to the MCWE composed of 16 elements and the BCWE composed of the BP-BC element. The dimensions of the MCWE were 280 mm × 150 mm (diameter × length), and it consists of 16 elements, each with dimensions of 40 mm × 150 mm, which were placed between the legs of the BP-BC RF coil. The dimensions of the BCWE were identical to those of the MCWE, and the BCWE was designed with a 16-leg BP-BC structure. The MCWE and the BCWE were tuned to 300 MHz using only passive elements without RF sources. The MCWE was configured with a 6.26 pF tuning capacitor, and the BCWE was configured with a 3.84 pF tuning capacitor. All wireless elements used in MCWE were tuned to a frequency of 300 MHz using tuning capacitors by applying an RF source, and only the RF source was removed. BCWE, like MCWE, was used by tuning to a frequency of 300 MHz in the presence of an RF source and then removing only the RF source. Since each capacitor applied to MCWE and BCWE was used by applying geometric phase information, the MCWE and BCWE were operated under circular polarization mode. For EM field simulations under ideal conditions, the Tx/Rx RF coils and WEs were made from perfect electric conductors.

The *sc*HPM consists of a segmented cylinder with an outer diameter of 315 mm, an inner diameter of 295 mm, and a length of 150 mm. The *sc*HPM was divided into 16 elements of the same size as that of the MCWE. The relative permittivity and loss tangent of the *sc*HPM were 300 and 0.05, respectively. The width of each RF coil element and the *sc*HPM was set to 40 mm so that the MCWE could be placed using the gap between the legs of the BP-BC RF coil. The *sc*HPM was also located between the Tx/Rx RF coil and the WE.

### 2.2. EM Field Analysis

To compare the EM field sensitivities and distributions generated by RF coil configurations under ideal conditions, an oil-based cylindrical phantom model with a diameter of 224 mm and a length of 150 mm was used. To analyze the EM field effects of RF coil configurations on the human body, EM field simulations were performed using a human head model (a HIFI head model with 17 tissue characteristics provided by Remcom, Inc., State College, PA, USA).

In general, the uniform phantoms used in the EM field simulation of MRI mainly use oil-based, water-based, and average phantoms (mean value of dielectric properties with white matter and gray matter in the human head). In the 7.0 T MRI simulation, the oil-based phantom consists primarily of dielectric properties with a conductivity 0 S·m^−1^ and a permittivity of 4. Furthermore, the water-based phantom using distilled water consists of dielectric properties with a conductivity 5 × 10^−5^ S·m^−1^ and a permittivity of 76.7. Moreover, the average phantom uses the average value of conductivity and permittivity of the white matter and gray matter in the human brain at a target frequency.

In this study, an oil-based cylindrical phantom was used, and EM field simulation using an oil-based cylindrical phantom was performed to evaluate the quantitative performance of the RF coil and verify the electromagnetic field distribution under ideal conditions. This was to minimize inhomogeneities, such as standing wave effects with shortened RF wavelength lengths in UHF MRI, distortions in peripheral region of the phantom due to high dielectric properties, and distortions of RF transmission and reception by shifted $B_{1}^{+}$/$B_{1}^{-}$ fields [91,92].

The EM field simulation calculations of the oil-based cylindrical phantom were performed for a total of 26,292,960 voxels (372 × 372 × 190 cells), while the EM field simulation calculations of the human head model were performed for a total of 66,022,560 voxels (522 × 372 × 340 cells). The voxel resolution of the EM field simulations was set to 1 mm^3^. For numerical analysis, EM field sensitivities and distributions composed of complex data matrices were analyzed in terms of the |$B_{1}^{+}$|, |$B_{1}^{-}$|, and |E| fields, and the SAR map. The FDTD complex data matrices of the EM field results were computed using MATLAB (version 2020a, Mathworks, Inc., Natick, MA, USA) for data analysis of the |$B_{1}^{+}$|, |$B_{1}^{-}$|, and |E| fields, and the SAR map.

EM field calculations were categorized into two groups: the unnormalized (|$B_{1}^{+}$|, |$B_{1}^{-}$|, and |E| fields) and normalized (|$B_{1}^{+}$| field and SAR map) cases. In the unnormalized case, we compared the EM field sensitivity changes for each combination of the Tx/Rx RF coil with the WE and the *sc*HPM under the same conditions, and the normalized case was used to predict the actual experiments when a 90° (π/2) RF pulse was induced. The total current applied to two types of RF coils in unnormalized condition was 1 A, and the equal current was applied to the combination of the two types of RF coils, as it was directly applied to the PEC without a capacitor at the position of the capacitor used to manufacture the actual RF coil. This method was applied to compare two types of RF coils under equal input RF power conditions, and the EM field generated by the equal amount of current applied to the conductor could be analyzed.

The unnormalized EM fields were compared using sensitivity-related factors and SD value-related factors as the relative differences, change rates, and capacity rates. The relative sensitivity differences and relative standard deviation (SD) value differences were calculated by comparing the 16-ch RF coil combination and the BP-BC RF coil combination. The sensitivity change ratio and SD value change ratio were calculated by comparing the Tx/Rx RF coil alone result with the result of applying the WE and *sc*HPM combinations. The capacity rate was used as a result of dividing the maximum EM field sensitivity value or the SD value of the 16-ch RF coil combinations by the maximum EM field sensitivity value or the SD value of the BP-BC RF coil combinations. The capability rate was a reference indicating how much the 16-ch RF coil combinations were relatively improved in terms of signal sensitivity and SD value compared to the BP-BC RF coil combination.

To calculate the normalized EM field, the efficiency of the unnormalized |$B_{1}^{+}$| field generated by the RF coil configurations was measured at the 3D center point values of the RF coil, and the calculated normalization coefficient (Norm-COEF) was applied to the unnormalized EM field. Specifically, the 3D center point values of the RF coil configurations in the normalized |$B_{1}^{+}$| field maps were calculated by assuming a flip angle of π/2; thus, the RF pulse was normalized at 1.957 μT [93,94]. By applying this calculated Norm-COEF to the unnormalized |$B_{1}^{+}$| field and the SAR map, normalized |$B_{1}^{+}$| field and SAR map analyses were performed assuming an actual 7.0 T MRI experiment.

The |$B_{1}$| field is composed of two circularly polarized components: the |$B_{1}^{+}$| and |$B_{1}^{-}$| fields. The components of the |$B_{1}^{+}$| and |$B_{1}^{-}$| fields are defined as $B_{1}^{+}$ and $B_{1}^{-}$ in the rotating frame, respectively. $B_{1}^{+}$ and $B_{1}^{-}$ can be defined as follows:

$$B_{1}^{+}=\left|\frac{\left(B_{1x}+iB_{1y}\right)}{\sqrt{2}}\;\right|,{\;B}_{1}^{-}=\left|\frac{\left(B_{1x}-iB_{1y}\right)}{\sqrt{2}}\;\right|$$

Here, $B_{1x}$ and $B_{1y}\;$are the $B_{1}$ components along the x- and y-axes, respectively.

The values of the unnormalized SAR map can be calculated as follows:

$$\mathrm{SAR}\left(r\right)=\frac{\sigma }{2\rho }{Ε}^{2}\propto \frac{dT}{dt},$$

where ${Ε}^{2}=Ε\cdot {Ε}^{*}$ denotes the squared magnitude of the induced E field, ρ=ρ(r) is the mass density (kg/m^3^), and T is the temperature (°K).

The whole-averaged SAR (mean SAR) and maximum SAR (max SAR) values were compared using the unaveraged SAR instead of the averaged SAR (1 g averaged SAR and 10 g averaged SAR) in normalized SAR maps. The mean SAR and max SAR values were calculated using mean and maximum values of unaveraged SAR in the entire tissue of the human head model, respectively. In addition, the unaveraged SAR was calculated by applying only the unaveraged SAR value of the entire human tissue region, excluding the massless region and the free space part of the human head model used.

## 3. Results and Discussion

**Figure 1:**
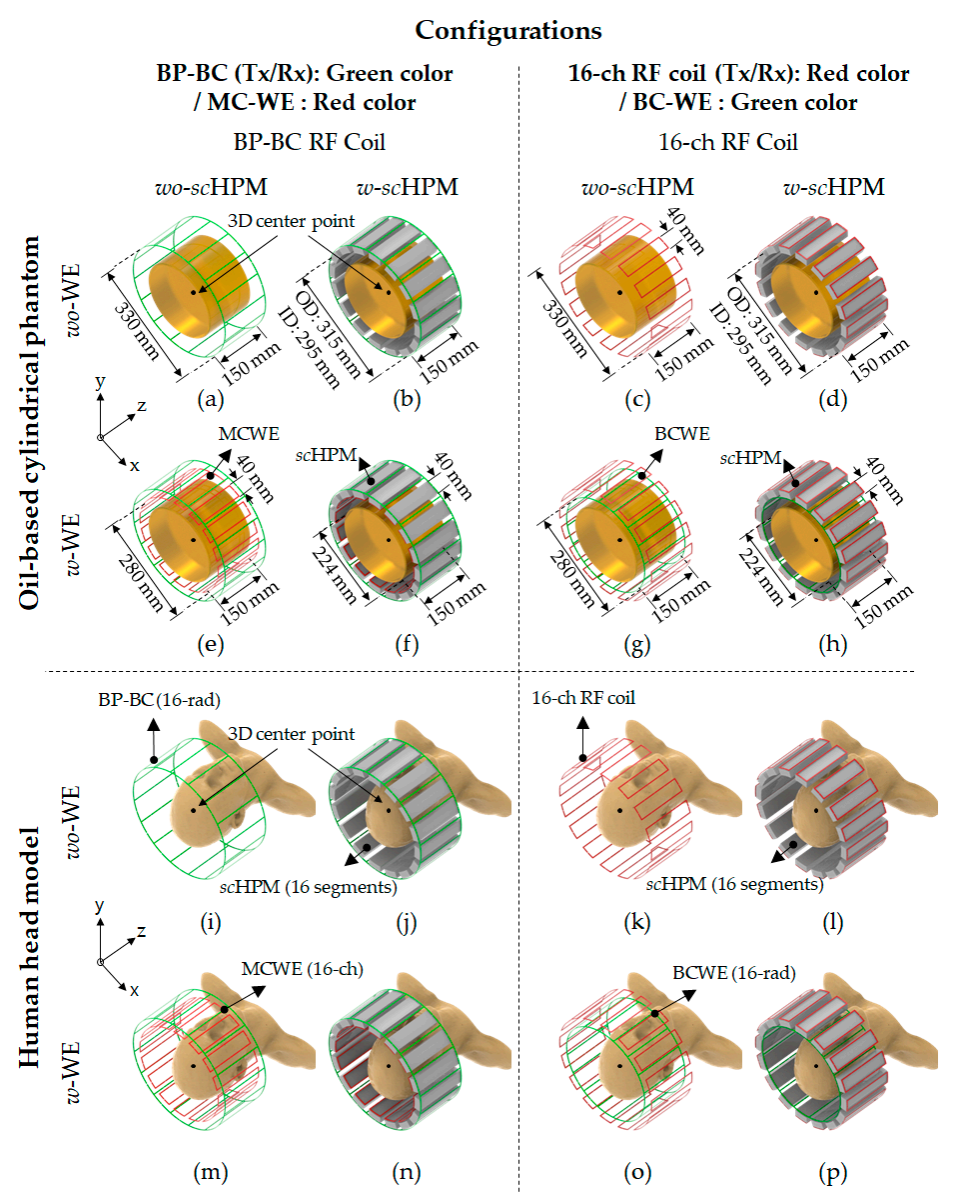
Configurations of Tx/Rx RF coil combinations for EM field simulations using the oil-based cylindrical phantom (**a**–**h**) and human head model (**i**–**p**): (**a**,i) BP-BC RF coil – *wo*-*sc*HPM – *wo*-MCWE; (**b**,j) BP-BC RF coil – *w*-*sc*HPM – *wo*-MCWE; (**c**,**k**) 16-ch RF coil – *wo*-*sc*HPM – *wo*-BCWE; (**d**,**j**) 16-ch RF coil – *w*-*sc*HPM – *wo*-BCWE; (**e**,**m**) BP-BC RF coil – *wo*-*sc*HPM – *w*-MCWE; (**f**,**l**) BP-BC RF coil – *w*-*sc*HPM – *w*-MCWE; (**g**,**o**) 16-ch RF coil – *wo*-*sc*HPM – *w*-BCWE; (**h**,**p**) 16-ch RF coil – *w*-*sc*HPM – *w*-BCWE.

**Figure 2:**
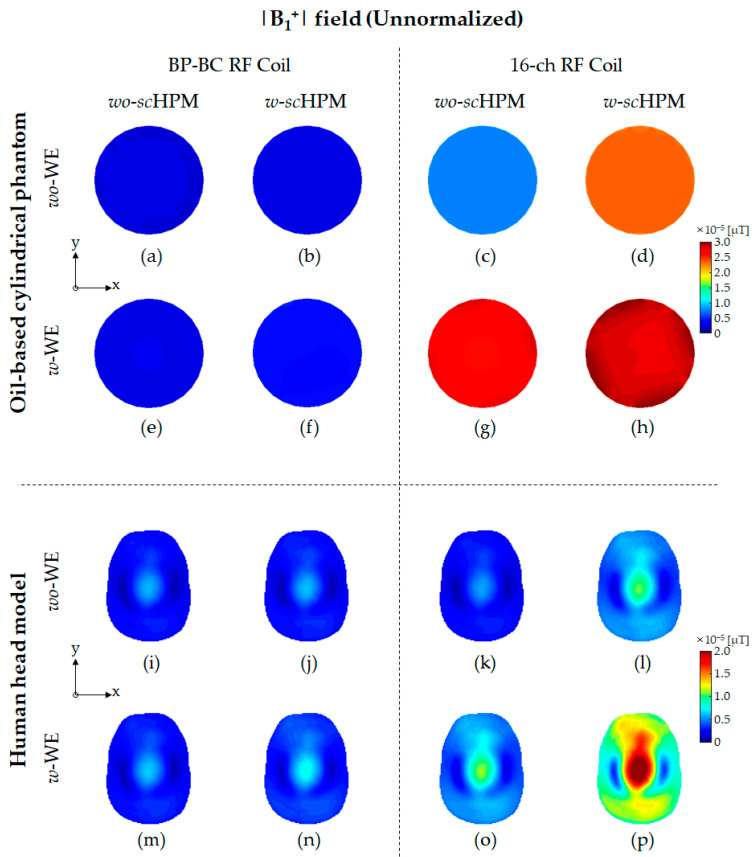
Unnormalized |$B_{1}^{+}$| field distributions using the oil-based cylindrical phantom (**a**–**h**) and human head model (**i**–**p**): (**a**,i) BP-BC RF coil – *wo*-*sc*HPM – *wo*-MCWE; (**b**,j) BP-BC RF coil – *w*-*sc*HPM – *wo*-MCWE; (**c**,**k**) 16-ch RF coil – *wo*-*sc*HPM – *wo*-BCWE; (**d**,**j**) 16-ch RF coil – *w*-*sc*HPM – *wo*-BCWE; (**e**,**m**) BP-BC RF coil – *wo*-*sc*HPM – *w*-MCWE; (**f**,**l**) BP-BC RF coil – *w*-*sc*HPM – *w*-MCWE; (**g**,**o**) 16-ch RF coil – *wo*-*sc*HPM – *w*-BCWE; (**h**,**p**) 16-ch RF coil – *w*-*sc*HPM – *w*-BCWE.

**Figure 3:**
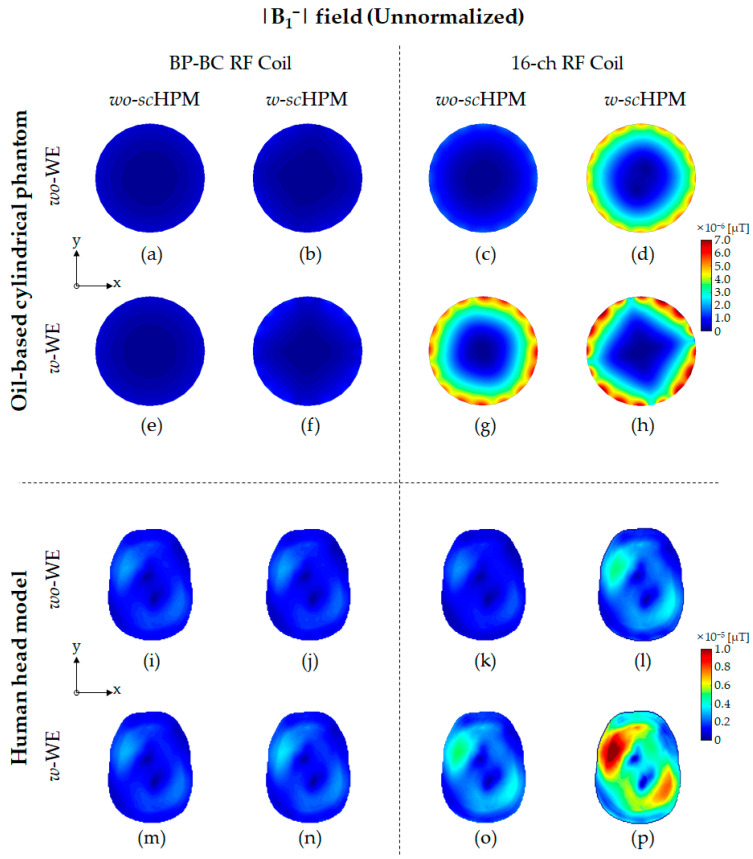
Unnormalized |$B_{1}^{-}$| field distributions using the oil-based cylindrical phantom (**a**–**h**) and human head model (**i**–**p**): (**a**,**i**) BP-BC RF coil – *wo*-*sc*HPM – *wo*-MCWE; (**b**,**j**) BP-BC RF coil – *w*-*sc*HPM – *wo*-MCWE; (**c**,**k**) 16-ch RF coil – *wo*-*sc*HPM – *wo*-BCWE; (**d**,**j**) 16-ch RF coil – *w*-*sc*HPM – *wo*-BCWE; (**e**,**m**) BP-BC RF coil – *wo*-*sc*HPM – *w*-MCWE; (**f**,**l**) BP-BC RF coil – *w*-*sc*HPM – *w*-MCWE; (**g**,**o**) 16-ch RF coil – *wo*-*sc*HPM – *w*-BCWE; (**h**,**p**) 16-ch RF coil – *w*-*sc*HPM – *w*-BCWE.

**Figure 4:**
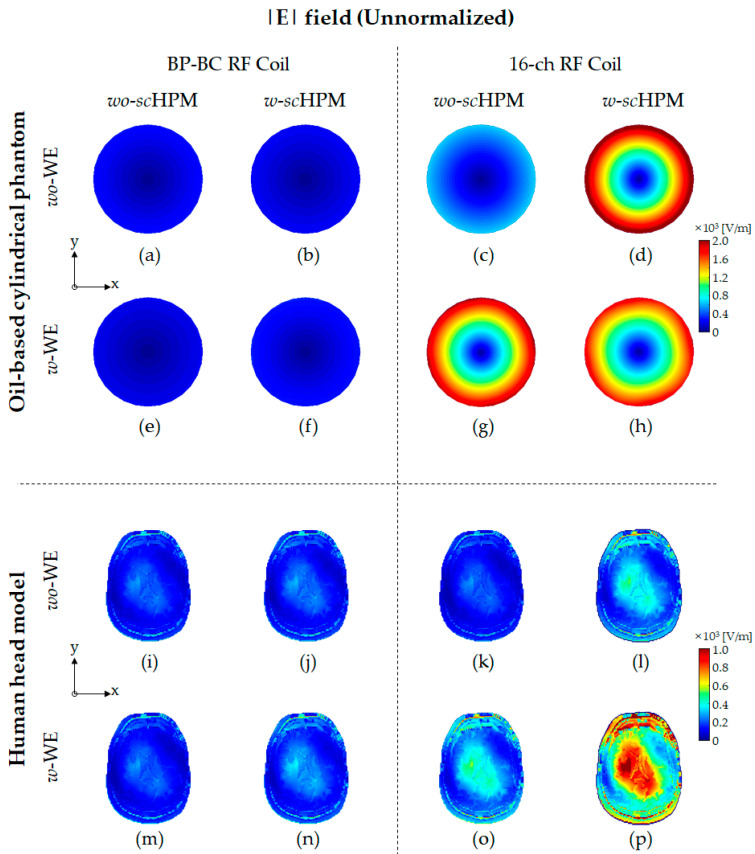
Unnormalized |E| field distributions using the oil-based cylindrical phantom (**a**–**h**) and human head model (**i**–**p**): (**a**,**i**) BP-BC RF coil – *wo*-*sc*HPM – *wo*-MCWE; (**b**,**j**) BP-BC RF coil – *w*-*sc*HPM – *wo*-MCWE; (**c**,**k**) 16-ch RF coil – *wo*-*sc*HPM – *wo*-BCWE; (**d**,**j**) 16-ch RF coil – *w*-*sc*HPM – *wo*-BCWE; (**e**,**m**) BP-BC RF coil – *wo*-*sc*HPM – *w*-MCWE; (**f**,**l**) BP-BC RF coil – *w*-*sc*HPM – *w*-MCWE; (**g**,**o**) 16-ch RF coil – *wo*-*sc*HPM – *w*-BCWE; (**h**,**p**) 16-ch RF coil – *w*-*sc*HPM – *w*-BCWE.

**Figure 5:**
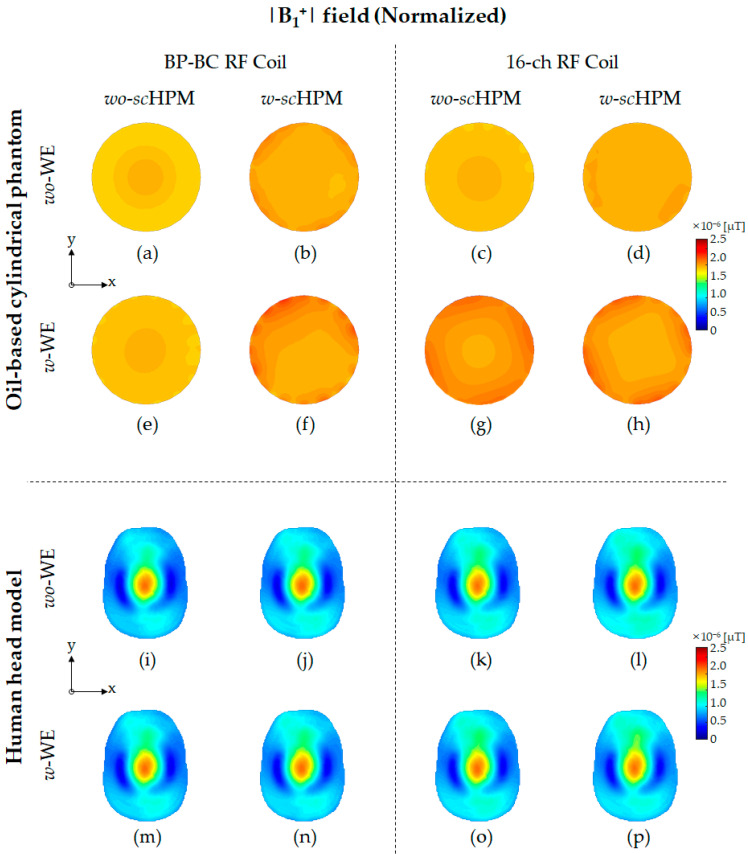
Normalized |$B_{1}^{+}$| field distributions using the oil-based cylindrical phantom (**a**–**h**) and human head model (**i**–**p**): (**a**,**i**) BP-BC RF coil – *wo*-*sc*HPM – *wo*-MCWE; (**b**,**j**) BP-BC RF coil – *w*-*sc*HPM – *wo*-MCWE; (**c**,**k**) 16-ch RF coil – *wo*-*sc*HPM – *wo*-BCWE; (**d**,**j**) 16-ch RF coil – *w*-*sc*HPM – *wo*-BCWE; (**e**,**m**) BP-BC RF coil – *wo*-*sc*HPM – *w*-MCWE; (**f**,**l**) BP-BC RF coil – *w*-*sc*HPM – *w*-MCWE; (**g**,**o**) 16-ch RF coil – *wo*-*sc*HPM – *w*-BCWE; (**h**,**p**) 16-ch RF coil – *w*-*sc*HPM – *w*-BCWE.

**Figure 6:**
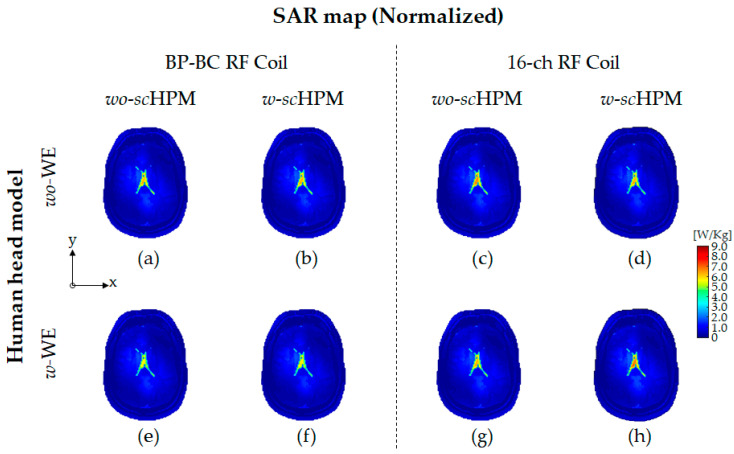
Normalized SAR maps constructed using Norm-COEF values: (**a**) BP-BC RF coil – *wo*-*sc*HPM – *wo*-MCWE; (**b**) BP-BC RF coil – *w*-*sc*HPM – *wo*-MCWE; (**c**) 16-ch RF coil – *wo*-*sc*HPM – *wo*-BCWE; (**d**) 16-ch RF coil – *w*-*sc*HPM – *wo*-BCWE; (**e**) BP-BC RF coil – *wo*-*sc*HPM – *w*-MCWE; (**f**) BP-BC RF coil – *w*-*sc*HPM – *w*-MCWE; (**g**) 16-ch RF coil – *wo*-*sc*HPM – *w*-BCWE; (**h**) 16-ch RF coil – *w*-*sc*HPM – *w*-BCWE.

To verify the effects of the *sc*HPM and the WE on the EM field generated by the BP-BC RF coil, we performed EM field simulations (Figure 1) using the BP-BC RF coil and the 16-ch RF coil with the *sc*HPM (*w*-*sc*HPM) or without the *sc*HPM (*wo*-*sc*HPM). Next, to evaluate the impact of the simultaneous use of the WE and the *sc*HPM, we compared the above configurations of the BP-BC RF coil with the WE (*w*-WE) or without the WE (*wo*-WE). The EM field results were simulated under unnormalized (Figure 2, Figure 3 and Figure 4) and normalized (Figure 5 and Figure 6) conditions.

Figure 1 shows the configurations of the numerical EM field simulation models. The EM field simulation models for the oil-based cylindrical phantom *wo*-WE involved the BP-BC RF coil alone (BP-BC RF coil – *wo*-s*c*HPM – *wo*-MCWE) (Figure 1a), the BP-BC RF coil with the *sc*HPM (BP-BC RF coil – *w*-s*c*HPM – *wo*-MCWE) (Figure 1b), the 16-ch RF coil alone (16-ch RF coil – *wo*-s*c*HPM – *wo*-BCWE) (Figure 1c), and the 16-ch RF coil with the *sc*HPM (16-ch RF coil – *w*-s*c*HPM – *wo*-BCWE) (Figure 1d), while those *w*-WE involved the BP-BC RF coil with the WE (BP-BC RF coil – *wo*-s*c*HPM – *w*-MCWE) (Figure 1e), the BP-BC RF coil with the s*c*HPM and the WE (BP-BC RF coil – *w*-s*c*HPM – *w*-MCWE) (Figure 1f), the 16-ch RF coil with the WE (16-ch RF coil – *wo*-s*c*HPM – *w*-BCWE) (Figure 1g), and the 16-ch RF coil with the *sc*HPM and the WE (16-ch RF coil – *w*-s*c*HPM – *w*-BCWE) (Figure 1h).

The EM fields were calculated numerically, and the sensitivity values were compared using the 3D center point values of the RF coil, as shown in Figure 1. For the quantitative analysis of the numerically calculated EM fields, we compared the values for the |$B_{1}^{+}$|, |$B_{1}^{-}$|, and |E| fields under the unnormalized condition. The 3D center point values (or max values) and standard deviation (SD) values for the unnormalized |$B_{1}^{+}$|, |$B_{1}^{-}$|, and |E| field sensitivities are shown in Table 1. We validated the Tx/Rx configurations by comparing the EM field sensitivities under the unnormalized conditions and compared the oil-based cylindrical phantom and human head model.

In the presence of the WE, not only the unnormalized |$B_{1}^{-}$| field improvement but also the unnormalized |$B_{1}^{+}$| field improvement was achieved, and in the 16-ch RF coil configuration with the BCWE, the unnormalized with the field improvement effect was much higher than that of the BP-BC RF coil configuration with the MCWE. This means that the proposed BCWE structure provided higher efficiency than the MCWE structure. In addition, the 16-ch RF coil configuration using *sc*HPM showed an excellent |$B_{1}^{+}$| efficiency improvement effect compared to the BP-BC RF coil configuration using *sc*HPM. This means that *sc*HPM applied to 16-ch RF coils can improve |$B_{1}^{+}$| efficiency more effectively (Figure 2d,l).

The unnormalized EM field simulation results were analyzed by calculating the unnormalized EM field sensitivity and SD values (listed in Table 1) of each Tx/Rx RF coil configuration. To evaluate their |$B_{1}^{+}$| field efficiency and |$B_{1}^{-}$| field sensitivity, the relative difference between the maximum sensitivities was calculated (listed in Table 1). The relative sensitivity difference was compared between the BP-BC RF coil and the 16-ch RF coil configurations (Case 1: comparing the BP-BC RF coil – *wo*-s*c*HPM – *wo*-MCWE and the 16-ch RF coil – *wo*-s*c*HPM – *wo*-BCWE; Case 2: comparing the BP-BC RF coil – *w*-s*c*HPM – *wo*-MCWE and the 16-ch RF coil – *w*-s*c*HPM – *wo*-BCWE; Case 3: comparing the BP-BC RF coil – *wo*-s*c*HPM – *w*-MCWE and the 16-ch RF coil – *wo*-s*c*HPM – *w*-BCWE; Case 4: comparing the BP-BC RF coil – *w*-s*c*HPM – *w*-MCWE and the 16-ch RF coil – *w*-s*c*HPM – *w*-BCWE).

In addition, to define the Tx/Rx RF coil combination that can provide optimal performance in terms of sensitivity and SD change ratios (listed in Table 2 and Table 3), we compared Tx/Rx RF coils – *wo*-s*c*HPM – *wo*-WE with Tx/Rx RF coil – *wo*-s*c*HPM – *w*-WE, Tx/Rx RF coil – *w*-s*c*HPM – *wo*-WE, and Tx/Rx RF coil – *w*-s*c*HPM – *w*-WE.

Figure 2 (see also Figures S1 and S2) shows the unnormalized |$B_{1}^{+}$| field distribution results using the oil-based cylindrical phantom and human head model. In the unnormalized |$B_{1}^{+}$| field results, the unnormalized |$B_{1}^{+}$| field distribution provided higher efficiency compared to the 16-ch RF coil alone. In particular, the sensitivity-related factors in all EM fields showed similar trends (listed in Table 1, Table 2 and Table 3).

The relative sensitivity differences in the unnormalized |$B_{1}^{+}$| field were evaluated using the oil-based cylindrical phantom and the human head model. In the oil-based cylindrical phantom results, the relative sensitivity differences in the unnormalized |$B_{1}^{+}$| field between the BP-BC RF coil and the 16-ch RF coil configurations were calculated to be approximately 3.004 times, 8.048 times, 9.894 times, and 7.072 times, respectively, under *wo*-WE/*wo*-*sc*HPM (Figure 2a,c), *wo*-WE/*w*-*sc*HPM (Figure 2b,d), *w*-WE/*wo*-*sc*HPM (Figure 2e,g), and *w*-WE/*w*-*sc*HPM (Figure 2f,h) conditions. In the human head model results, the relative sensitivity differences in the unnormalized |$B_{1}^{+}$| field between the BP-BC RF coil and the 16-ch RF coil configurations were calculated to be approximately 0.942 times, 1.754 times, 1.746 times, and 3.056 times, respectively, under *wo*-WE/*wo*-*sc*HPM (Figure 2i,k), *wo*-WE/*w*-*sc*HPM (Figure 2j,l), *w*-WE/*wo*-*sc*HPM (Figure 2m,o), and *w*-WE/*w*-*sc*HPM (Figure 2n,p) conditions.

The sensitivity change ratios of the unnormalized |$B_{1}^{+}$| field using the oil-based cylindrical phantom were calculated as approximately 1.178 times (for BP-BC RF coil configurations comparing Figure 2a and Figure 2b), 1.150 times (for BP-BC RF coil configurations comparing Figure 2a and Figure 2e), 1.794 times (for BP-BC RF coil configurations comparing Figure 2a and Figure 2f), 3.156 times (for 16-ch RF coil configurations comparing Figure 2c and Figure 2d), 3.787 times (for 16-ch RF coil configurations comparing Figure 2c and Figure 2g), and 4.224 times (for 16-ch RF coil configurations comparing Figure 2c and Figure 2h). In the human head model results, the sensitivity change ratios of the unnormalized |$B_{1}^{+}$| field were calculated as approximately 1.036 times (for BP-BC RF coil configurations comparing Figure 2i and Figure 2j), 1.083 times (for BP-BC RF coil configurations comparing Figure 2i and Figure 2m), 1.328 times (for BP-BC RF coil configurations comparing Figure 2i and Figure 2n), 1.928 times (for 16-ch RF coil configurations comparing Figure 2k and Figure 2l), 2.007 times (for 16-ch RF coil configurations comparing Figure 2k and Figure 2o), and 4.309 times (for 16-ch RF coil configurations comparing Figure 2k and Figure 2p).

The sensitivity capability rates of the unnormalized |$B_{1}^{+}$| field using the oil-based cylindrical phantom were calculated as 267.909% (comparing the BP-BC RF coil − *w*-s*c*HPM – *wo*-MCWE and the 16-ch RF coil – *w*-s*c*HPM – *wo*-BCWE), 329.368% (comparing the BP-BC RF coil – *wo*-s*c*HPM – *w*-MCWE and the 16-ch RF coil – *wo*-s*c*HPM – *w*-BCWE), and 235.423% (comparing the BP-BC RF coil – *w*-s*c*HPM – *w*-MCWE and the 16-ch RF coil – *w*-s*c*HPM – *w*-BCWE). The sensitivity capability rates of the unnormalized |$B_{1}^{+}$| field using the human head model were calculated as 186.132% (comparing the BP-BC RF coil – *w*-s*c*HPM – *wo*-MCWE and the 16-ch RF coil – *w*-s*c*HPM – *wo*-BCWE), 185.282% (comparing the BP-BC RF coil – *wo*-s*c*HPM – *w*-MCWE and the 16-ch RF coil – *wo*-s*c*HPM – *w*-BCWE), and 324.391% (comparing the BP-BC RF coil – *w*-s*c*HPM – *w*-MCWE and the 16-ch RF coil – *w*-s*c*HPM – *w*-BCWE).

The sensitivity-related factors of the unnormalized |$B_{1}^{+}$| field results showed remarkably similar tendencies, indicating improved sensitivity in the 16-ch RF coil configurations compared to the BP-BC RF coil configurations. In particular, the sensitivity change ratio and the sensitivity capability rate showed extremely similar tendencies.

The SD value relative differences in the unnormalized |$B_{1}^{+}$| field were evaluated using the oil-based cylindrical phantom and human head model. In the oil-based cylindrical phantom results, the relative sensitivity differences between the BP-BC RF coil and the 16-ch RF coil configurations were calculated to be approximately 1.750 times, 2.250 times, 19.333 times, and 6.455 times, respectively, under *wo*-WE/*wo*-*sc*HPM (Figure 2a,c), *wo*-WE/*w*-*sc*HPM (Figure 2b,d), *w*-WE/*wo*-*sc*HPM (Figure 2e,g), and *w*-WE/*w*-*sc*HPM (Figure 2f,h) conditions. In the human head model results, the relative SD value differences in the unnormalized |$B_{1}$| field between the BP-BC RF coil and the 16-ch RF coil configurations were calculated to be approximately 0.925 times, 1.755 times, 1.730 times, and 3.092 times, respectively, under *wo*-WE/*wo*-*sc*HPM (Figure 2i,k), *wo*-WE/*w*-*sc*HPM (Figure 2j,l), *w*-WE/*wo*-*sc*HPM (Figure 2m,o), and *w*-WE/*w*-*sc*HPM (Figure 2n,p) conditions.

The SD value change ratios of the unnormalized |$B_{1}^{+}$| field using the oil-based cylindrical phantom were calculated as approximately 1.000 times (for BP-BC RF coil configurations comparing Figure 2a and Figure 2b), 0.750 times (for BP-BC RF coil configurations comparing Figure 2a and Figure 2e), 2.750 times (for BP-BC RF coil configurations comparing Figure 2a and Figure 2f), 1.286 times (for 16-ch RF coil configurations comparing Figure 2c and Figure 2d), 8.286 times (for 16-ch RF coil configurations comparing Figure 2c and Figure 2g), and 10.143 times (for 16-ch RF coil configurations comparing Figure 2c and Figure 2h). In the human head model results, the SD value change ratios of the unnormalized |$B_{1}^{+}$|field were calculated as approximately 1.011 times (for BP-BC RF coil configurations comparing Figure 2i and Figure 2j), 1.075 times (for BP-BC RF coil configurations comparing Figure 2i and Figure 2m), 1.290 times (for BP-BC RF coil configurations comparing Figure 2i and Figure 2n), 1.919 times (for 16-ch RF coil configurations comparing Figure 2k and Figure 2l), 2.012 times (for 16-ch RF coil configurations comparing Figure 2k and Figure 2o), and 4.314 times (for 16-ch RF coil configurations comparing Figure 2k and Figure 2p).

The SD value capability rates of the unnormalized |$B_{1}^{+}$| field using the oil-based cylindrical phantom were calculated as 128.571% (comparing the BP-BC RF coil – *w*-s*c*HPM – *wo*-MCWE and the 16-ch RF coil – *w*-s*c*HPM – *wo*-BCWE), 1104.762% (comparing the BP-BC RF coil – *wo*-s*c*HPM – *w*-MCWE and the 16-ch RF coil – *wo*-s*c*HPM – *w*-BCWE), and 368.312% (comparing the BP-BC RF coil – *w*-s*c*HPM – *w*-MCWE and the 16-ch RF coil – *w*-s*c*HPM – *w*-BCWE). The sensitivity capability rates using the human head model were calculated as 189.819% (comparing the BP-BC RF coil – *w*-s*c*HPM – *wo*-MCWE and the 16-ch RF coil – *w*-s*c*HPM – *wo*-BCWE), 187.081% (comparing the BP-BC RF coil – *wo*-s*c*HPM – *w*-MCWE and the 16-ch RF coil – *wo*-s*c*HPM – *w*-BCWE), and 334.331% (comparing the BP-BC RF coil – *w*-s*c*HPM – *w*-MCWE and the 16-ch RF coil – *w*-s*c*HPM – *w*-BCWE).

As can be seen from the unnormalized |$B_{1}^{+}$| field results, the sensitivity value, sensitivity change ratio, and SD value change ratio of the unnormalized |$B_{1}^{+}$| field were greatly influenced by Tx/Rx RF coil configurations with the *sc*HPM and the WE. In terms of the SD value change ratio of the unnormalized |$B_{1}^{+}$| field, the SD value change ratio of the 16-ch RF coil alone – *wo*-BCWE compared with those of 16-ch RF coil + *sc*HPM, the 16-ch RF coil alone – *w*-BCWE, and the 16-ch RF coil + *sc*HPM – *w*-BCWE using the oil-based cylindrical phantom showed very contradictory results compared to the SD value change ratios of other EM fields. As shown in Figure 2, in the unnormalized |$B_{1}^{+}$| field results, the relative differences between the oil-based cylindrical phantom and the human head model results proved that the *sc*HPM *w*-BCWE applied to the 16-ch RF coil was more sensitive to dielectric properties. The extremely different SD value change ratio between the |$B_{1}^{+}$| field results of the oil-based cylindrical phantom and the human head model was that the oil-based cylindrical phantom was relatively closer to the 16-RF coil configuration applied *sc*HPM and BCWE than the human head model. In addition, compared to the human head model, there was a large difference in the rate of change in SD values due to the dielectric properties of the oil-based phantom. However, in the 16-ch RF coil configurations, the SD values of the unnormalized |$B_{1}^{+}$| fields changed relatively significantly, while the SD values of unnormalized |$B_{1}^{-}$| and |E| fields showed a change similar to the sensitivity change ratio of the unnormalized |$B_{1}^{-}$| and |E| field.

However, unlike the sensitivity-related factors of the unnormalized |$B_{1}^{+}$| field results, the SD value-related factors of the unnormalized |$B_{1}^{+}$| field results increased dramatically due to the WE in the oil-based cylindrical phantom results. This rapid increase in the SD value-related factors was expected to intensify due to the BCWE, which consists of volume shapes that adhere to the cylindrical phantom, and its low dielectric properties. On the other hand, in the human head model results, it was confirmed that the sensitivity-related and SD value-related factors of the unnormalized |$B_{1}^{+}$| field showed a similar tendency. A sharp increase in the SD value of the BCWE was observed using the oil-based cylindrical phantom model, but a sharp change in the SD value-related factors could not be observed in the human head model. Based on the oil-based cylindrical phantom results, a rapid change in the SD value-related factors was observed in the 16-ch RF coil configuration with the BCWE, but the advantage of the sensitivity-related factors increased in terms of RF power. Thus, it is worth recommending the use of the 16-ch RF coil – *wo*-s*c*HPM – *w*-BCWE and the 16-ch RF coil – *w*-s*c*HPM – *w*-BCWE.

Figure 3 (see also Figures S3 and S4) shows the unnormalized |$B_{1}^{-}$| field distribution results using the oil-based cylindrical phantom and human head model. From the unnormalized |$B_{1}^{-}$| field results shown in Figure 3, the combinations of *w*-*sc*HPM and *wo/w*-BCWE (Figure 3d–h) allowed us to observe extreme changes in |$B_{1}^{-}$| field sensitivity in the periphery region of the oil-based cylindrical phantom, as it was located close enough to the BCWE and the *sc*HPM. Similar abnormal unnormalized |$B_{1}^{-}$| field patterns in the periphery region were not observed in the human head model results, but the unnormalized |$B_{1}^{-}$| field penetration into the deep region of the human head model was confirmed. The unnormalized |$B_{1}^{-}$| field distribution also provided higher sensitivity compared to the 16-ch RF coil alone, depending on how WE and *sc*HPM combinations were applied.

The relative sensitivity differences in the unnormalized |$B_{1}^{-}$| field were evaluated using the oil-based cylindrical phantom and human head model. In the oil-based cylindrical phantom results, the relative sensitivity differences in the unnormalized |$B_{1}^{-}$| field between the BP-BC RF coil and the 16-ch RF coil configurations were calculated to be approximately 3.120 times, 7.563 times, 11.362 times, and 6.125 times, respectively, under *wo*-WE/*wo*-*sc*HPM (Figure 3a,c), *wo*-WE/*w*-*sc*HPM (Figure 3b,d), *w*-WE/*wo*-*sc*HPM (Figure 3e,g), and *w*-WE/*w*-*sc*HPM (Figure 3f,h) conditions. In the human head model results, the relative sensitivity differences in the unnormalized |$B_{1}^{-}$| field between the BP-BC RF coil and the 16-ch RF coil configurations were calculated to be approximately 0.945 times, 1.784 times, 1.740 times, and 3.124 times, respectively, under *wo*-WE/*wo*-*sc*HPM (Figure 3i,k), *wo*-WE/*w*-*sc*HPM (Figure 3j,l), *w*-WE/*wo*-*sc*HPM (Figure 3m,o), and *w*-WE/*w*-*sc*HPM (Figure 3n,p) conditions.

The sensitivity change ratios of the unnormalized |$B_{1}^{-}$| field using the oil-based cylindrical phantom were calculated as approximately 1.420 times (BP-BC RF coil configurations—comparison of Figure 3a and Figure 3b), 1.160 times (BP-BC RF coil configurations—comparison of Figure 3a and Figure 3e), 2.560 times (BP-BC RF coil configurations—comparison of Figure 3a and Figure 3f), 3.442 times (16-ch RF coil configurations—comparison of Figure 3c and Figure 3d), 4.224 times (16-ch RF coil configurations—comparison of Figure 3c and Figure 3g), and 5.026 times (16-ch RF coil configurations—comparison of Figure 3c and Figure 3h). In the human head model results, the sensitivity change ratios of the unnormalized |$B_{1}^{-}$| field were calculated as approximately 1.007 times (BP-BC RF coil configurations—comparison of Figure 3i and Figure 3j), 1.077 times (BP-BC RF coil configurations—comparison of Figure 3i and Figure 3m), 1.277 times (BP-BC RF coil configurations—comparison of Figure 3i and Figure 3n), 1.902 times (16-ch RF coil configurations—comparison of Figure 3k and Figure 3l), 1.984 times (16-ch RF coil configurations—comparison of Figure 3k and Figure 3o), and 4.223 times (16-ch RF coil configurations—comparison of Figure 3k and Figure 3p).

The sensitivity capability rates of the unnormalized |$B_{1}^{-}$| field using the oil-based cylindrical phantom were calculated as 242.416% (comparing the BP-BC RF coil – *w*-s*c*HPM – *wo*-MCWE and the 16-ch RF coil – *w*-s*c*HPM – *wo*-BCWE), 364.169% (comparing the BP-BC RF coil – *wo*-s*c*HPM – *w*-MCWE and the 16-ch RF coil – *wo*-s*c*HPM – *w*-BCWE), and 196.314% (comparing the BP-BC RF coil – *w*-s*c*HPM – *w*-MCWE and the 16-ch RF coil – *w*-s*c*HPM – *w*-BCWE). The sensitivity capability rates of the unnormalized |$B_{1}$| field using the human head model were calculated as 188.841% (comparing the BP-BC RF coil – *w*-s*c*HPM – *wo*-MCWE and the 16-ch RF coil – *w*-s*c*HPM – *wo*-BCWE), 184.166% (comparing the BP-BC RF coil – *wo*-s*c*HPM – *w*-MCWE and the 16-ch RF coil – *wo*-s*c*HPM – *w*-BCWE), and 330.734% (comparing the BP-BC RF coil – *w*-s*c*HPM – *w*-MCWE and the 16-ch RF coil – *w*-s*c*HPM – *w*-BCWE).

The sensitivity-related factors of the unnormalized |$B_{1}^{-}$| field results indicated significantly improved sensitivity in the 16-ch RF coil configurations compared to the BP-BC RF coil configurations. Particularly, in the oil-based cylindrical phantom results, sensitivity-related factors were detected at the position where the 16-ch RF coil and the *sc*HPM structure were disposed in the periphery region of the oil phantom. As shown in Figure 3g, the unnormalized |$B_{1}^{-}$| field sensitivity rapidly increased due to the influence of the BCWE located inside the 16-ch RF coil. These results confirmed that the unnormalized |$B_{1}^{-}$| field improvement effect of the 16-ch RF coil with the BCWE was significantly better than that of the BP-BC RF coil with the MCWE, and that the BCWE structure was much more effective than the MCWE structure when the WE was applied to the Tx/Rx RF coil.

The relative SD value differences of the unnormalized |$B_{1}^{-}$| field were evaluated using the oil-based cylindrical phantom and human head model. In the oil-based cylindrical phantom results, the relative sensitivity differences between the BP-BC RF coil and the 16-ch RF coil configurations were calculated to be approximately 2.929 times, 8.563 times, 11.000 times, and 6.800 times, respectively, under *wo*-WE/*wo*-*sc*HPM (Figure 3a,c), *wo*-WE/*w*-*sc*HPM (Figure 3b,d), *w*-WE/*wo*-*sc*HPM (Figure 3e,g), and *w*-WE/*w*-*sc*HPM (Figure 3f,h) conditions. In the human head model results, the relative SD value differences in the unnormalized |$B_{1}^{-}$| field between the BP-BC RF coil and the 16-ch RF coil configurations were calculated to be approximately 0.959 times, 1.796 times, 1.750 times, and 3.183 times, respectively, under *wo*-WE/*wo*-*sc*HPM (Figure 3i,k), *wo*-WE/*w*-*sc*HPM (Figure 3j,l), *w*-WE/*wo*-*sc*HPM (Figure 3m,o), and *w*-WE/*w*-*sc*HPM (Figure 3n,p) conditions.

The SD value change ratios of the unnormalized |$B_{1}^{-}$| field using the oil-based cylindrical phantom were calculated as approximately 1.143 times (BP-BC RF coil configurations—comparison of Figure 3a and Figure 3b), 1.000 times (BP-BC RF coil configurations—comparison of Figure 3a and Figure 3e), 1.786 times (BP-BC RF coil configurations—comparison of Figure 3a and Figure 3f), 3.341 times (16-ch RF coil configurations—comparison of Figure 3c and Figure 3d), 3.756 times (16-ch RF coil configurations—comparison of Figure 3c and Figure 3g), and 4.146 times (16-ch RF coil configurations—comparison of Figure 3c and Figure 3h). In the human head model results, the SD value change ratios of the unnormalized |$B_{1}^{-}$| field were calculated as approximately 1.021 times (BP-BC RF coil configurations—comparison of Figure 3i and Figure 3j),1.083 times (BP-BC RF coil configurations—comparison of Figure 3i and Figure 3m), 1.250 times (BP-BC RF coil configurations—comparison of Figure 3i and Figure 3n), 1.913 times (16-ch RF coil configurations—comparison of Figure 3k and Figure 3l), 1.978 times (16-ch RF coil configurations—comparison of Figure 3k and Figure 3o), and 4.152 times (16-ch RF coil configurations—comparison of Figure 3k and Figure 3p).

The SD value capability rates of the unnormalized |$B_{1}^{-}$| field using the oil-based cylindrical phantom was calculated as 128.571% (comparing the BP-BC RF coil – *w*-s*c*HPM – *wo*-MCWE and the 16-ch RF coil – *w*-s*c*HPM – *wo*-BCWE), 1104.762% (comparing the BP-BC RF coil – *wo*-s*c*HPM – *w*-MCWE and the 16-ch RF coil – *wo*-s*c*HPM – *w*-BCWE), and 368.312% (comparing the BP-BC RF coil – *w*-s*c*HPM – *w*-MCWE and the 16-ch RF coil – *w*-s*c*HPM – *w*-BCWE). The sensitivity capability rates using the human head model were calculated as 189.819% (comparing the BP-BC RF coil – *w*-s*c*HPM – *wo*-MCWE and the 16-ch RF coil – *w*-s*c*HPM – *wo*-BCWE), 187.081% (comparing the BP-BC RF coil – *wo*-s*c*HPM – *w*-MCWE and the 16-ch RF coil – *wo*-s*c*HPM – *w*-BCWE), and 334.331% (comparing the BP-BC RF coil – *w*-s*c*HPM – *w*-MCWE and the 16-ch RF coil – *w*-s*c*HPM – *w*-BCWE).

As shown in Table 2 and Table 3, the sensitivity-related and SD value-related factors of the unnormalized |$B_{1}^{-}$| field showed similar trends. In other words, it was confirmed that the sensitivity-related factors and SD value-related factors of the unnormalized |$B_{1}^{-}$| field increased with the application of the *sc*HPM and the WE, and in the case of the 16-ch RF coil with the *sc*HPM and the BCWE (in Figure 3h,p), the sensitivity-related and SD value-related factors of the unnormalized |$B_{1}^{-}$| field improved simultaneously.

Figure 4 (see also Figures S5 and S6) shows the unnormalized |E| field distribution results using the oil-based cylindrical phantom and human head model. The strong unnormalized |E| field concentration was observed in the peripheral region of the oil-based cylindrical phantom and the central region of the human head model. The unnormalized |E| field distribution was similar to the unnormalized |$B_{1}^{-}$| field distribution, and it was confirmed that the unnormalized |E| field was more concentrated in the Tx/Rx RF coil configuration with the *sc*HPM and the WE than in the Tx/Rx RF coil alone. When comparing the 16-ch RF coil – *w*-s*c*HPM – *wo*-BCWE (Figure 4d,l) and the 16-ch RF coil – *wo*-s*c*HPM – *w*-BCWE (Figure 4g,o) results, it should be noted that the 16-ch RF coil – *wo*-s*c*HPM – *w*-BCWE (Figure 4g,o) had a stronger unnormalized |E| field concentration than the 16-ch RF coil – *w*-s*c*HPM – *wo*- BCWE (Figure 4d,l). These results were obtained from the unnormalized |E| field concentration between the 16-ch RF coil and the BCWE. In the 16-ch RF coil – *w*-s*c*HPM – *w*-BCWE (Figure 4h,p), the unnormalized |E| field concentration among the 16-ch RF coil, *sc*HPM, and BCWE became stronger.

The relative sensitivity differences in the unnormalized |E| field were evaluated using the oil-based cylindrical phantom and human head model. In the oil-based cylindrical phantom results, the relative sensitivity differences in the unnormalized |E| field between the BP-BC RF coil and the 16-ch RF coil configurations were calculated to be approximately 2.475 times, 10.665 times, 8.908 times, and 6.411 times, respectively, under *wo*-WE/*wo*-*sc*HPM (Figure 4a,c), *wo*-WE/*w*-*sc*HPM (Figure 4b,d), *w*-WE/*wo*-*sc*HPM (Figure 4e,g), and *w*-WE/*w*-*sc*HPM (Figure 4f,h) conditions. In the human head model results, the relative sensitivity differences in the unnormalized |E| field between the BP-BC RF coil and the 16-ch RF coil configurations were calculated to be approximately 0.829 times, 1.668 times, 1.663 times, and 2.967 times, respectively, under *wo*-WE/*wo*-*sc*HPM (Figure 4i,k), *wo*-WE/*w*-*sc*HPM (Figure 4j,l), *w*-WE/*wo*-*sc*HPM (Figure 4m,o), and *w*-WE/*w*-*sc*HPM (Figure 4n,p) conditions.

The sensitivity change ratios of the unnormalized |E| field using the oil-based cylindrical phantom were calculated as approximately 0.779 times (BP-BC RF coil configurations—comparison of Figure 4a and Figure 4b), 0.862 times (BP-BC RF coil configurations—comparison of Figure 4a and Figure 4e), 1.139 times (BP-BC RF coil configurations—comparison of Figure 4a and Figure 4f), 3.360 times (16-ch RF coil configurations—comparison of Figure 4c and Figure 4d), 3.103 times (16-ch RF coil configurations—comparison of Figure 4c and Figure 4g), and 2.950 times (16-ch RF coil configurations—comparison of Figure 4c and Figure 4h). In the human head model results, the sensitivity change ratios of the unnormalized |E| field were calculated as approximately 0.978 times (BP-BC RF coil configurations—comparison of Figure 4i and Figure 4j), 1.017 times (BP-BC RF coil configurations—comparison of Figure 4i and Figure 4m), 1.263 times (BP-BC RF coil configurations—comparison of Figure 4i and Figure 4n), 1.967 times (16-ch RF coil configurations—comparison of Figure 4k and Figure 4l), 2.040 times (16-ch RF coil configurations—comparison of Figure 4k and Figure 4o), and 4.518 times (16-ch RF coil configurations—comparison of Figure 4k and Figure 4p).

The sensitivity capability rates of the unnormalized |E| field using the oil-based cylindrical phantom were calculated as 430.947% (comparing the BP-BC RF coil – *w*-s*c*HPM – *wo*-MCWE and the 16-ch RF coil – *w*-s*c*HPM – *wo*-BCWE), 359.932% (comparing the BP-BC RF coil – *wo*-s*c*HPM – *w*-MCWE and the 16-ch RF coil – *wo*-s*c*HPM – *w*-BCWE), and 259.046% (comparing the BP-BC RF coil – *w*-s*c*HPM – *w*-MCWE and the 16-ch RF coil – *w*-s*c*HPM – *w*-BCWE). The sensitivity capability rates of the unnormalized |$B_{1}$| field using the human head model were calculated as 201.203% (comparing the BP-BC RF coil – *w*-s*c*HPM – *wo*-MCWE and the 16-ch RF coil – *w*-s*c*HPM – *wo*-BCWE), 200.523% (comparing the BP-BC RF coil – *wo*-s*c*HPM – *w*-MCWE and the 16-ch RF coil – *wo*-s*c*HPM – *w*-BCWE), and 357.844% (comparing the BP-BC RF coil – *w*-s*c*HPM – *w*-MCWE and the 16-ch RF coil – *w*-s*c*HPM – *w*-BCWE).

The sensitivity-related factors of the unnormalized |E| field results tended to contradict the results of other unnormalized EM fields (|$B_{1}^{+}$| and |$B_{1}^{-}$| fields), except for the unnormalized |E| field in the 16-ch RF coil configuration in the oil-based cylindrical phantom results. In particular, in the oil-based cylindrical phantom results, unlike the human head model results, the sensitivity change ratio of the unnormalized |E| field was the highest for 16-ch RF coil – *w*-s*c*HPM – *wo*-BCWE and the lowest for 16-ch RF coil – *w*-s*c*HPM – *w*-BCWE. These results showed that uniform phantoms with low dielectric properties, located very uniformly and close to the *sc*HPM, such as the oil-based cylindrical phantom, can increase the concentration of the unnormalized |E| field by the *sc*HPM. In addition, it was confirmed that the unnormalized |E| field concentration was higher in the case of the BP-BC RF coil configuration with the WE (BP-BC RF coil – *wo*-s*c*HPM – *w*-MCWE), whereas the unnormalized |E| field concentration was higher in the periphery region of the oil-based cylindrical phantom owing to the *sc*HPM than in the 16-ch RF coil configuration (16-ch RF coil – *wo*-s*c*HPM – *w*-BCWE).

The relative SD value differences in the unnormalized |E| field were evaluated using the oil-based cylindrical phantom and human head model. In the oil-based cylindrical phantom results, the relative SD value differences between the BP-BC RF coil and the 16-ch RF coil configurations were calculated to be approximately 2.561 times, 11.626 times, 0.910 times, and 1.747 times, respectively, under *wo*-WE/*wo*-*sc*HPM (Figure 4a,c), *wo*-WE/*w*-*sc*HPM (Figure 4b,d), *w*-WE/*wo*-*sc*HPM (Figure 4e,g), and *w*-WE/*w*-*sc*HPM (Figure 4f,h) conditions. In the human head model results, the relative SD value differences in the unnormalized |E| field between the BP-BC RF coil and the 16-ch RF coil configurations were calculated to be approximately 0.910 times, 1.747 times, 1.743 times, and 3.083 times, respectively, under *wo*-WE/*wo*-*sc*HPM (Figure 4i,k), *wo*-WE/*w*-*sc*HPM (Figure 4j,l), *w*-WE/*wo*-*sc*HPM (Figure 4m,o), and *w*-WE/*w*-*sc*HPM (Figure 4n,p) conditions.

The SD value change ratios of the unnormalized |E| field using the oil-based cylindrical phantom were calculated as approximately 0.738 times (BP-BC RF coil configurations—comparison of Figure 4a and Figure 4b), 0.835 times (BP-BC RF coil configurations—comparison of Figure 4a and Figure 4e), 1.066 times (BP-BC RF coil configurations—comparison of Figure 4a and Figure 4f), 3.352 times (16-ch RF coil configurations—comparison of Figure 4c and Figure 4d), 3.007 times (16-ch RF coil configurations—comparison of Figure 4c and Figure 4g), and 2.741 times (16-ch RF coil configurations—comparison of Figure 4c and Figure 5h). In human head model results, the SD value change ratios of the unnormalized |E| field were calculated as approximately 1.003 times (BP-BC RF coil configurations—comparison of Figure 4i and Figure 4j), 1.056 times (BP-BC RF coil configurations—comparison of Figure 4i and Figure 4m), 1.270 times (BP-BC RF coil configurations—comparison of Figure 4i and Figure 4n), 1.926 times (16-ch RF coil configurations—comparison of Figure 4k and Figure 4l), 2.023 times (16-ch RF coil configurations—comparison of Figure 4k and Figure 4o), and 4.303 times (16-ch RF coil configurations—comparison of Figure 4k and Figure 4p).

The SD value capability rates of the unnormalized |E| field using the oil-based cylindrical phantom were calculated as 453.999% (comparing the BP-BC RF coil – *w*-s*c*HPM – *wo*-MCWE and the 16-ch RF coil – *w*-s*c*HPM – *wo*-BCWE), 360.129% (comparing the BP-BC RF coil – *wo*-s*c*HPM – *w*-MCWE and the 16-ch RF coil – *wo*-s*c*HPM – *w*-BCWE), and 257.186% (comparing the BP-BC RF coil – *w*-s*c*HPM – *w*-MCWE and the 16-ch RF coil – *w*-s*c*HPM – *w*-BCWE). The sensitivity capability rates using the human head model were calculated as 192.056% (comparing the BP-BC RF coil – *w*-s*c*HPM – *wo*-MCWE and the 16-ch RF coil – *w*-s*c*HPM – *wo*-BCWE), 191.606% (comparing the BP-BC RF coil – *wo*-s*c*HPM – *w*-MCWE and the 16-ch RF coil – *wo*-s*c*HPM – *w*-BCWE), and 338.888% (comparing the BP-BC RF coil – *w*-s*c*HPM – *w*-MCWE and the 16-ch RF coil – *w*-s*c*HPM – *w*-BCWE).

For the oil-based cylindrical phantom, the SD value-related and sensitivity-related factors of the unnormalized |E| field tended to be very similar. In contrast to the BP-BC RF coil – *wo*-s*c*HPM – *w*-MCWE, which represents the highest SD value distribution, the highest SD value distribution appeared in the 16-ch RF coil – *w*-s*c*HPM – *wo*-BCWE, followed by the 16-ch RF coil – *wo*-s*c*HPM – *w*-BCWE and the 16-ch RF coil – *w*-s*c*HPM – *w*-BCWE. As shown in Table 2 and Table 3, the unnormalized |$B_{1}^{+}$| field efficiency and unnormalized |$B_{1}^{-}$| field sensitivity improvement were confirmed by applying *sc*HPM with the same dimension as the 16-ch RF coil and volume-type BCWE compared to BP-BC RF coil configurations. In addition, it was observed that the sensitivity-related factors and the SD value-related factors in the unnormalized |E| field improve to similar levels as those in other EM fields (such as the unnormalized |$B_{1}^{+}$| field and the unnormalized |$B_{1}^{-}$| field). However, this is not because of the reduction of EM field efficiency by the 16-ch RF coil with the *sc*HPM and the BCWE but the improved sensitivity of the entire EM field. The EM field efficiency improvement was higher in the 16-ch RF coil configuration.

In addition, to access the uniformity of the EM fields, the coefficient of variation (CV) was calculated using the SD values (listed in Table 1) and mean values (listed in Table 4) of EM fields. The CV is defined as the ratio of the SD value to the mean value. It shows the extent of variability in relation to the mean of the EM field results. As listed in Table 4, the mean values were calculated using the non-zero values of the EM field, and the change tendency of the mean values according to the RF coil configurations was similar to that of the SD values (listed in Table 1).

When comparing uniformity in terms of CV, the 16-ch RF coil configurations in terms of |$B_{1}^{+}$| and |$B_{1}^{-}$| field uniformity showed no significant difference from the BP-BC RF coil configurations and provided similar CV. This shows that in terms of |$B_{1}^{+}$| and |$B_{1}^{-}$| field uniformity, the 16-ch RF coil configurations can improve |$B_{1}^{+}$| field efficiency and |$B_{1}^{-}$| field sensitivity without degrading the |$B_{1}^{+}$| and |$B_{1}^{-}$| field uniformity compared to BP-BC RF coil configurations.

However, in terms of the |E| field uniformity, the 16-ch RF coil configuration was observed to reduce relative uniformity in oil-based cylindrical phantom results that were not observable in human head model results. According to the oil-based cylindrical phantom results, the CV values of the |E| field increased due to the low dielectric properties of the oil-based cylindrical phantom used, and the |E| field uniformity could not be confirmed in the human head model results.

Unlike the unnormalized EM field analysis results, such as |$B_{1}^{+}$|, |$B_{1}^{-}$|, and |E| fields, the normalized |$B_{1}^{+}$| field (Figure 5) and the SAR map (Figure 6) were calculated applying a 90° RF pulse to the RF coils. To calculate the normalized EM field using the unnormalized EM field results, we used a method to calculate necessary normalized values and then applied the computed normalized values to the SAR map and the |$B_{1}^{+}$| field subjected to a 90° RF pulse. The Norm-COEF values were calculated for the BP-BC RF coil and the 16-ch RF coil configurations, respectively, as listed in Table 5.

As shown in Table 5 and Figure 5, the Norm-COEF and normalized |$B_{1}^{+}$| fields were calculated and analyzed using the oil-based cylindrical phantom and human head model. In the oil-based cylindrical phantom results, the Norm-COEF values of the BP-BC RF coil configurations were calculated as 0.793 (BP-BC RF coil – *wo*-s*c*HPM – *wo*-MCWE), 0.723 (BP-BC RF coil – *w*-s*c*HPM – *wo*-MCWE), 0.689 (BP-BC RF coil – *wo*-s*c*HPM – *w*-MCWE), and 0.521 (BP-BC RF coil – *w*-s*c*HPM – *w*-MCWE). The Norm-COEF values of the 16-ch RF coil configurations were calculated as 0.264 (16-ch RF coil – *wo*-s*c*HPM – *wo*-BCWE), 0.071 (16-ch RF coil – *w*-s*c*HPM – *wo*-BCWE), 0.085 (16-ch RF coil – *wo*-s*c*HPM – *w*-BCWE), and 0.076 (16-ch RF coil – *w*-s*c*HPM – *w*-BCWE).

In the human head model results, the Norm-COEF values of the BP-BC RF coil configurations were calculated as 0.362 (BP-BC RF coil – *wo*-s*c*HPM – *wo*-MCWE), 0.352 (BP-BC RF coil – *w*-s*c*HPM – *wo*-MCWE), 0.334 (BP-BC RF coil – *wo*-s*c*HPM – *w*-MCWE), and 0.273 (BP-BC RF coil – *w*-s*c*HPM – *w*-MCWE). The Norm-COEF values of the 16-ch RF coil configurations were calculated as 0.383 (16-ch RF coil – *wo*-s*c*HPM – *wo*-BCWE), 0.087 (16-ch RF coil – *w*-s*c*HPM – *wo*-BCWE), 0.192 (16-ch RF coil – *wo*-s*c*HPM – *w*-BCWE), and 0.199 (16-ch RF coil – *w*-s*c*HPM – *w*-BCWE).

The normalized |$B_{1}^{+}$| fields after the application of the Norm-COEFs (listed in Table 5) were calculated, as shown in Figure 5 (also see Figures S7 and S8). The Norm-COEF of the 16-ch RF coil with the *sc*HPM or WE was significantly lower compared to the BP-BC RF coil with the *sc*HPM or WE. In other words, applying the *sc*HPM or WE to the 16-ch RF coil excited an ^1^H by a 90° RF pulse at the center point of the target object with less RF power than applying the *sc*HPM or WE to the BP-BC RF coil. In terms of Norm-COEF, the 16-ch RF coil – *w*-s*c*HPM – *w*-BCWE provided 3.718 and 4.402 times lower RF power requirements in the oil-based cylindrical phantom and human head model, respectively, compared to 16-ch RF coil – *wo*-s*c*HPM – *wo*-BCWE. The 16-ch RF coil – *w*-s*c*HPM – *w*-BCWE also provided 11.169 and 4.161 times lower RF power requirements in the oil-based cylindrical phantom and human head model, respectively, compared to the BP-BC RF – *wo*-s*c*HPM – *wo*-MCWE.

In the normalized |$B_{1}^{+}$| field results using oil-based cylindrical phantom and human head models, the center values of the BP-BC RF coil and the 16-ch RF coil were both normalized to values when a 90° RF pulse was applied. For the comparison of uniformity of 16-ch RF coil combinations and BP-BC RF coil combinations, the mean value, SD value, and CV of the normalized |$B_{1}^{+}$| field were evaluated as shown in Figure 5 and Table 6. The 16-ch RF coil configurations produced high unnormalized |$B_{1}^{+}$| field efficiency (as listed in Table 5) without degrading the uniformity of the normalized |$B_{1}^{+}$| field distribution compared to the BP-BC RF coil configurations (as listed in Table 6). These results were equally observed in the human head model results, and it was confirmed that the 16-ch RF coil configuration provided a more sensitive and uniform normalized |$B_{1}^{+}$| field distribution.

The SAR maps after the application of the Norm-COEFs were calculated, as shown in Figure 6 (also see Figures S9 and S10) and Table 7. Since the unnormalized SAR maps involve the SAR results without the consideration of the transmission RF power, more quantitative SAR maps were analyzed by calculating the normalized SAR maps by applying a 90° RF pulse. The mean and max SAR values are listed in Table 7. In the SAR maps of the BP-BC RF coil and the 16-ch RF coil configurations, it was not possible to confirm the rapid change in the SAR map distribution, as shown in Figure 6.

In the BP-BC RF coil configuration results, the mean SAR values were calculated as 0.209 W/kg (BP-BC RF coil – *wo*-s*c*HPM – *wo*-MCWE), 0.215 W/kg (BP-BC RF coil – *w*-s*c*HPM – *wo*-MCWE), 0.216 W/kg (BP-BC RF coil – *wo*-s*c*HPM – *w*-MCWE), and 0.211 W/kg (BP-BC RF coil – *w*-s*c*HPM – *w*-MCWE). The mean SAR values of the 16-ch RF coil configurations were calculated as 0.249 W/kg (16-ch RF coil – *wo*-s*c*HPM – *wo*-BCWE), 0.238 W/kg (16-ch RF coil – *w*-s*c*HPM – *wo*-BCWE), 0.243 W/kg (16-ch RF coil – *wo*-s*c*HPM – *w*-BCWE), and 0.227 W/kg (16-ch RF coil – *w*-s*c*HPM – *w*-BCWE).

The max SAR values of the BP-BC RF coil configurations were calculated as 8.203 W/kg (BP-BC RF coil – *wo*-s*c*HPM – *wo*-MCWE), 8.461 W/kg (BP-BC RF coil – *w*-s*c*HPM – *wo*-MCWE), 8.376 W/kg (BP-BC RF coil – *wo*-s*c*HPM – *w*-MCWE), and 8.248 W/kg (BP-BC RF coil – *w*-s*c*HPM – *w*-MCWE), and the max SAR values of the 16-ch RF coil configurations were calculated as 8.496 W/kg (16-ch RF coil – *wo*-s*c*HPM – *wo*-BCWE), 8.472 W/kg (16-ch RF coil – *w*-s*c*HPM – *wo*-BCWE), 8.284 W/kg (16-ch RF coil – *wo*-s*c*HPM – *w*-BCWE), and 8.552 W/kg (16-ch RF coil – *w*-s*c*HPM – *w*-BCWE).

Ironically, the results of the normalized SAR maps and SAR values showed no significant changes. Since the 16-ch RF coil with the *sc*HPM and WE provided higher |$B_{1}^{+}$| field efficiency, the distribution of SAR maps was almost unchanged compared to those without the *sc*HPM and the WE, as the RF coil could be excited by 90° using low RF power.

To summarize the unnormalized EM field simulation results, the unnormalized |$B_{1}^{+}$| field sensitivity improvement rate between the 16-ch RF coil – *w*-s*c*HPM – *w*-MCWE (improved 422.237% in the oil-based cylindrical phantom and 430.866% in the human head model compared to the 16-ch RF coil alone) and the BP-BC RF coil – *w*-s*c*HPM – *w*-MCWE (improved 179.352% in the oil-based cylindrical phantom and 132.823% in the human head model compared to the BP-BC RF coil alone) was 235.424% based on the oil-based cylindrical phantom result and 324.391% based on the human head model.

Similarly, the unnormalized |$B_{1}^{-}$| field sensitivity improvement rate between the 16-ch RF coil – *w*-s*c*HPM – *w*-MCWE (improved 502.564% in the oil-based cylindrical phantom and 422.266% in the human head model compared to the 16-ch RF coil alone) and the BP-BC RF coil – *w*-s*c*HPM – *w*-MCWE (improved 256.000% in oil-based cylindrical phantom and 127.675% in the human head model compared to the BP-BC RF coil alone) was 196.314% and 330.735% based on the oil-based cylindrical phantom and the human head model, respectively.

Moreover, the unnormalized |E| field sensitivity improvement rate between the 16-ch RF coil – *w*-s*c*HPM – *w*-MCWE (improved 126.252% in the oil-based cylindrical phantom and 451.784% in the human head model compared to the 16-ch RF coil alone) and the BP-BC RF coil – *w*-s*c*HPM – *w*-MCWE (improved 113.897% in the oil-based cylindrical phantom and 295.045% in the human head model compared to the BP-BC RF coil alone) was found to 110.848% and 153.124%, respectively, using the oil-based cylindrical phantom and the human head model.

In the unnormalized EM field simulation results, the unnormalized |$B_{1}^{+}$| and |$B_{1}^{-}$| field sensitivity improvement rates of the 16-ch RF coil with the *sc*HPM and the BCWE were substantial and showed surprisingly similar tendencies. In particular, the 16-ch RF coil consisting of the same dimensions as the *sc*HPM was used as a Tx/Rx RF coil to significantly improve the |$B_{1}^{+}$| field sensitivity. In addition, it was confirmed that the sensitivity of the |$B_{1}^{-}$| field was greatly improved by applying the BCWE, which provided a volume structure, compared to the MCWE.

Moreover, the 16-ch RF coil with the *sc*HPM and the BCWE showed little change in the unnormalized |E| field sensitivity improvement rate. The |E| field sensitivity improvement rate was calculated under unnormalized conditions, but when applying the Norm-COEF to an |E| field distribution, the 16-ch RF coil with the *sc*HPM and the BCWE could exhibit a lower |E| field distribution. In addition, these results were also applied to the SAR maps, and the normalized SAR maps were also measured with a 16-ch RF coil without any difference.

In the normalized EM field simulation results, the normalized |$B_{1}^{+}$| field in the 16-ch RF coil with the *sc*HPM and the BCWE showed a high unnormalized |$B_{1}^{+}$| field efficiency without degrading the uniformity of the normalized |$B_{1}^{+}$| field distribution compared to the BP-BC RF coil with the *sc*HPM and the MCWE. In particular, the results of the human head model confirmed improved |$B_{1}^{+}$| field efficiency in a wider area from the 16-ch RF coil with the *sc*HPM and the BCWE to the frontal lobe. As a result, it was confirmed that the 16-ch RF coil with the *sc*HPM and the BCWE had better |$B_{1}^{+}$| field efficiency and uniformity than the BP-BC RF coil with the *sc*HPM and the MCWE and could significantly improve |$B_{1}^{+}$| field efficiency without degrading |$B_{1}^{+}$| field uniformity.

For the SAR map with the applied Norm-COEFs, the 16-ch RF coil combination with the BCWE and the *sc*HPM provided RF safety in terms of sufficiently reduced SAR due to the relatively low RF power applied to the 16-ch RF coil. Under the normalized condition, both the |$B_{1}^{+}$| field efficiency and |$B_{1}^{-}$| field sensitivity of the 16-ch RF coil – *w*-*sc*HPM – *w*-BCWE increased dramatically compared to the BP-BC RF coil – *w*-*sc*HPM – *w*-MCWE, providing SAR distributions similar to those of the BP-BC RC coil or the 16-ch RF coil alone.

The unnormalized (|$B_{1}^{+}$|, |$B_{1}^{-}$|, and |E| fields) and normalized EM field results (normalized |$B_{1}^{+}$| field and SAR maps) of the 16-ch RF coil with the *sc*HPM and the BCWE and the BP-BC RF coil with *sc*HPM and the MCWE were compared in the *x*–*z* and *y*–*z* planes, as well as in the *x*–*y* plane, and the results are provided in the Supplementary Materials (Figures S1−S10).

Here, we describe the results of the EM field simulations of the 16-ch RF coil with the *sc*HPM and the BCWE, which was intended to improve |$B_{1}^{+}$| field efficiency and |$B_{1}^{-}$| field sensitivity. The *sc*HPM structure has proved to be optimal and has clear limitations in further structure improvement [47]. The BP-BC RF coil proposed in our previous work has been widely used in MRI because it provides a highly uniform $B_{1}$ field distribution, but a decrease in RF wavelength after increasing the $B_{0}$ field strength reduces the $B_{1}$ field uniformity, which is a critical weakness for volume coils, such as BP-BC RF coils. In addition, the MCWE used as the WE was configured as a multichannel considering interference with the *sc*HPM, but there were limitations in providing sufficient |$B_{1}^{+}$| and |$B_{1}^{-}$| field improvement capabilities.

To address these problems, we decided to change the role of the BP-BC RF coil and the MCWE. The 16-ch RF coil, which provides a more uniform |$B_{1}^{+}$| and |$B_{1}^{-}$| field distribution with a higher sensitivity than the BC RF coil in UHF MRI, was changed and applied to a Tx/Rx coil, and the BP-BC RF coil was used to improve the |$B_{1}^{-}$| field sensitivity in the MCWE.

As a result, the 16-ch RF coil consisting of the same dimensions as the *sc*HPM was able to dramatically improve the |$B_{1}^{+}$| field efficiency, and the BCWE consisting of the BC RF coil was also able to significantly improve the |$B_{1}^{-}$| field sensitivity. Thus, to improve the limited $B_{1}$ field sensitivity due to the reduced RF transceiver efficiency and sensitivity in UHF MRI, the proposed combination of RF coil configurations can be adopted using a BCWE for improving |$B_{1}^{-}$| field sensitivity and an *sc*HPM for enhancing |$B_{1}^{+}$| field efficiency.

In this study, the |$B_{1}^{+}$| field efficiency of the 16-ch RF coil with the *sc*HPM was significantly improved compared to the BP-BC RF coil with the *sc*HPM by configuring the *sc*HPM structure to fit the size of the 16-ch RF coil and increasing the energy density without distorting the RF transmit direction. Furthermore, by replacing the MCWE with the BCWE, higher |$B_{1}^{-}$| field efficiency was ensured.

The synergy between the BCWE and *sc*HPM configurations allowed the |$B_{1}^{+}$| and |$B_{1}^{-}$| fields in the 16-ch RF coil to dramatically enhance the Tx/Rx efficiency and sensitivity compared to BP-BC RF coil – *w*-s*c*HPM – *w*-MCWE. Improvements in Tx/Rx efficiency and sensitivity could consequently lead to |$B_{1}$| field improvement, which can be seen in Figures S11−S13.

Our numerical EM field calculations also showed that the proposed combination of the 16-ch RF coil with the *sc*HPM and the BCWE provided enhanced |$B_{1}^{+}$| field efficiency and |$B_{1}^{-}$| field sensitivity in the human head model. The SAR values of the proposed combination indicate its suitability for UHF MRI.

To discuss a single limitation in this study, we were unable to obtain MR images for the human head using the proposed 16-ch RF coil – *w*-s*c*HPM – *w*-BCWE. The MRI system currently in possession is a 7.0 T MRI scanner (Siemens, Magnetom, Germany), which is currently operated jointly in hospitals and laboratories. For the 7.0 T MRI system currently in operation, it is essential to obtain an Institutional Review Board (IRB) for human application with research equipment permit, not clinical-only equipment.

Acquisition of an Instrumental Review Board (IRB) is essential for human imaging using 7.0 T MRI systems for clinical and research purposes. However, the 7.0 T MRI used for clinical and research purposes is strictly prohibited for obtaining human images with artifacts such as *sc*HPM and BCWE inserted inside the main magnet bore. For this reason, it may be difficult to obtain human head images of the 16-ch RF coil – *w*-s*c*HPM – *w*-BCWE using 7.0 T MRI systems for future human imaging, so we plan to implement the 16-ch RF coil – *w*-s*c*HPM – *w*-BCWE using a pre-clinical MRI system.

## 4. Conclusions

The proposed new combination of the Tx/Rx RF coil configuration using an *sc*HPM and a WE was tried to simultaneously improve |$B_{1}^{+}$| field efficiency and |$B_{1}^{-}$| field sensitivity in UHF MRI. In our previous study, we proposed an RF coil combination for the 7.0 T MRI system that provided improved |$B_{1}^{+}$| field efficiency and |$B_{1}^{-}$| field sensitivity over the BP-BC RF coil combination with the *sc*HPM and the MCWE.

Since the *sc*HPM structure has already been determined to be the optimal structure, a method for simultaneously improving |$B_{1}^{+}$| field efficiency and |$B_{1}^{-}$| field sensitivity by optimizing the structures of RF coils and the WE was developed. As a result, the propagation direction of the RF transmission power through the *sc*HPM was further clarified using a 16-ch RF coil as a Tx/Rx RF coil, and a volumetric WE (BCWE) was proposed to maximize the efficiency of |$B_{1}^{-}$| field sensitivity. Through these processes, the 16-ch RF coil with the *sc*HPM and the BCWE was proposed as a new RF coil combination to simultaneously improve the $B_{1}^{+}$ field efficiency and the $B_{1}^{-}$ field sensitivity.

The EM field simulations were performed to verify the effects of the proposed configurations using numerical calculations. The configuration of the 16-ch RF coil combination with the *sc*HPM and the BCWE was modified to switch the roles of the BP-BC RF coil and the MCWE. The 16-ch RF coil combination with the *sc*HPM and the BCWE was compared to the BP-BC RF coil combination with the *sc*HPM and the MCWE. The structures of the *sc*HPM and the WE (BCWE and MCWE) were designed to match the propagation direction of the RF wave generated by the Tx/Rx RF coils (16-ch RF coil and BP-BC RF coil) and the positions of both the *sc*HPM and the WE (BCWE and MCWE). Specifically, the design and dimensions of both the *sc*HPM and the WE (BCWE and MCWE) were such that they could be located between the elements of the 16-ch RF coil and the legs of the BC coil.

The proposed 16-ch RF coil combination with the *sc*HPM and the BCWE dramatically improved both the |$B_{1}^{+}$| field efficiency and |$B_{1}^{-}$| field sensitivity of the 16-ch RF coil in UHF MRI. The |$B_{1}^{+}$| field efficiency and |$B_{1}^{-}$| field sensitivity of 16-ch RF coil combination with the *sc*HPM and the BCWE provided significant performance improvements over the BP-BC RF coil combination with the *sc*HPM and the MCWE proposed in the previous work. According to these results, the proposed RF coil configuration can improve the performance of 16-ch RF coils limited by low RF power and SAR issues in UHF MRI (above 7.0 T).

Nevertheless, the application of the 16-ch RF coil with the *sc*HPM and the BCWE to the actual clinical 7.0 T MRI failed, and we could not perform it experimentally to verify with the EM field simulation results. MR image acquisition using unauthorized devices such as *sc*HPMs and BCWEs is strictly prohibited. Due to strict MRI safety regulations, actual experiments using the *sc*HPM and the BCWE could not be performed. However, through the findings demonstrated, we confirmed the possibility of *sc*HPM and BCWE use to simultaneously enhance |$B_{1}^{+}$| field efficiency and |$B_{1}^{-}$| field sensitivity.

In our future work, a study on the combination of the 16-ch RF coil with the *sc*HPM and the MCWE will be conducted using numerical EM field simulations. To conduct a study on the optimized MCWE location, we will study the effects of the alignment and tilt angle of 16-ch RF coil, *sc*HPM, and MCWE.

## Figures and Tables

**Table 1:** Maximum and SD values of unnormalized EM field simulation results (|$B_{1}^{+}$|, |$B_{1}^{-}$|, and |E| fields) using the oil-based cylindrical phantom and human head model.

			BP-BC RF Coil *wo*-*sc*HPM	BP-BC RF Coil *w*-*sc*HPM	16-ch RF Coil *wo*-*sc*HPM	16-ch RF Coil *w*-*sc*HPM	
	Max values of the unnormalized |$B_{1}^{+}$| field						×10^−5^ (μT)
Oil-based cylindrical phantom		*wo*-WE	0.247	0.291	0.742	2.342	
		*w*-WE	0.284	0.443	2.810	3.133	
Human head model		*wo*-WE	0.588	0.609	0.554	1.068	
		*w*-WE	0.637	0.781	1.112	2.387	
	SD values of the unnormalized |$B_{1}^{+}$| field						×10^−5^ (μT)
Oil-based cylindrical phantom		*wo*-WE	0.004	0.004	0.007	0.009	
		*w*-WE	0.003	0.011	0.058	0.071	
Human head model		*wo*-WE	0.093	0.094	0.086	0.165	
		*w*-WE	0.100	0.120	0.173	0.371	
	Max values of the unnormalized |$B_{1}^{-}$| field						×10^−5^ (μT)
Oil-based cylindrical phantom		*wo*-WE	0.050	0.071	0.156	0.537	
		*w*-WE	0.058	0.128	0.659	0.784	
Human head model		*wo*-WE	0.271	0.273	0.256	0.487	
		*w*-WE	0.292	0.346	0.508	1.081	
	SD values of the unnormalized |$B_{1}^{-}$| field						×10^−5^ (μT)
Oil-based cylindrical phantom		*wo*-WE	0.014	0.016	0.041	0.137	
		*w*-WE	0.014	0.025	0.154	0.170	
Human head model		*wo*-WE	0.048	0.049	0.046	0.088	
		*w*-WE	0.052	0.060	0.091	0.191	
	Max values of the unnormalized |E| field						(V/m)
Oil-based cylindrical phantom		*wo*-WE	260.257	202.869	644.104	2163.678	
		*w*-WE	224.388	296.424	1998.820	1900.393	
Human head model		*wo*-WE	727.420	711.303	603.219	1186.803	
		*w*-WE	740.012	918.380	1230.528	2725.248	
	SD values of the unnormalized |E| field						(V/m)
Oil-based cylindrical phantom		*wo*-WE	60.182	44.438	154.113	516.632	
		*w*-WE	50.242	64.141	463.342	422.434	
Human head model		*wo*-WE	62.877	63.044	57.199	110.145	
		*w*-WE	66.373	79.834	115.689	246.113	

**Table 2:** Unnormalized EM field (|$B_{1}$|, |$B_{1}^{+}$|, |$B_{1}^{-}$|, and |E| fields) sensitivity change ratios for *sc*HPM – *wo*-WE, *w*-WE, and *w*-*sc*HPM – *w*-WE compared to the Tx/Rx RF coil alone using oil-based cylindrical phantom and human head model.

			BP-BC RF Coil *w*-*sc*HPM – *wo*-WE	16-ch RF Coil *w*-*sc*HPM – *wo*-WE	Capability Rate	
	Sensitivity change ratio of the unnormalized |$B_{1}^{+}$| field					(%)
Oil-based cylindrical phantom		*w*-*sc*HPM	117.814	315.633	267.909	
		*w*-WE	114.980	378.706	329.368	
		*w*-*sc*HPM – *w*-WE	132.823	430.866	324.391	
Human head model		*w*-*sc*HPM	103.571	192.780	186.132	
		*w*-WE	108.333	200.722	182.282	
		*w*-*sc*HPM – *w*-WE	132.823	430.866	324.391	
	Sensitivity change ratio of the unnormalized |$B_{1}^{-}$| field					(%)
Oil-based cylindrical phantom		*w*-*sc*HPM	142.000	344.231	242.146	
		*w*-WE	116.000	422.436	364.169	
		*w*-*sc*HPM – *w*-WE	256.000	502.564	196.314	
Human head model		*w*-*sc*HPM	100.738	190.234	188.841	
		*w*-WE	107.749	198.438	184.166	
		*w*-*sc*HPM – *w*-WE	127.675	422.266	330.734	
	Sensitivity change ratio of the unnormalized |E| field					(%)
Oil-based cylindrical phantom		*w*-*sc*HPM	77.950	335.921	430.947	
		*w*-WE	86.218	310.326	359.932	
		*w*-*sc*HPM – *w*-WE	113.897	295.044	259.046	
Human head model		*w*-*sc*HPM	97.784	196.745	201.203	
		*w*-WE	101.731	203.994	200.522	
		*w*-*sc*HPM – *w*-WE	126.252	451.784	357.844	

**Table 3:** SD change ratios of the unnormalized EM field (|$B_{1}^{+}$|, |$B_{1}^{-}$|, and |E| fields) for *sc*HPM – *wo*-WE, *w*-WE, and *sc*HPM – *w*-WE compared to the Tx/Rx RF coil alone using the oil-based cylindrical phantom and human head model.

			BP-BC RF Coil *w*-*sc*HPM – *wo*-WE	16-ch RF Coil *w*-*sc*HPM – *wo*-WE	Capability Rate	
	SD change ratio of the unnormalized |$B_{1}^{+}$| field					(%)
Oil-based cylindrical phantom		*w*-*sc*HPM	100.000	128.571	128.571	
		*w*-WE	75.000	828.571	1104.762	
		*w*-*sc*HPM – *w*-WE	275.000	1014.286	368.831	
Human head model		*w*-*sc*HPM	101.075	191.861	189.819	
		*w*-WE	107.527	201.163	187.081	
		*w*-*sc*HPM – *w*-WE	129.032	431.395	334.331	
	SD change ratio of the unnormalized |$B_{1}^{-}$| field					(%)
Oil-based cylindrical phantom		*w*-*sc*HPM	114.286	334.146	292.378	
		*w*-WE	100.000	375.601	375.610	
		*w*-*sc*HPM – *w*-WE	178.571	414.634	232.195	
Human head model		*w*-*sc*HPM	102.083	191.304	187.400	
		*w*-WE	108.333	197.826	182.609	
		*w*-*sc*HPM – *w*-WE	125.000	415.217	332.174	
	SD change ratio of the unnormalized |E| field					(%)
Oil-based cylindrical phantom		*w*-*sc*HPM	73.839	335.229	453.999	
		*w*-WE	83.484	300.650	360.129	
		*w*-*sc*HPM – *w*-WE	106.579	274.106	257.186	
Human head model		*w*-*sc*HPM	100.266	192.566	192.056	
		*w*-WE	105.556	202.258	191.606	
		*w*-*sc*HPM – *w*-WE	126.968	430.279	338.888	

**Table 4:** Coefficient of variation (SD/mean) in unnormalized EM field simulation results (|$B_{1}^{+}$|, |$B_{1}^{-}$|, and |E| fields) using the oil-based cylindrical phantom and human head model.

			BP-BC RF Coil *wo*-*sc*HPM	BP-BC RF Coil *w*-*sc*HPM	16-ch RF Coil *wo*-*sc*HPM	16-ch RF Coil *w*-*sc*HPM	
	Mean values of the unnormalized |$B_{1}^{+}$| field						×10^−5^ (μT)
Oil-based cylindrical phantom		*wo*-WE	0.237	0.271	0.722	2.827	
		*w*-WE	0.276	0.383	2.312	2.656	
Human head model		*wo*-WE	0.251	0.269	0.251	0.499	
		*w*-WE	0.280	0.350	0.512	1.112	
	Mean values of the unnormalized |$B_{1}^{-}$| field						×10^−5^ (μT)
Oil-based cylindrical phantom		*wo*-WE	0.026	0.024	0.063	0.248	
		*w*-WE	0.023	0.038	0.234	0.262	
Human head model		*wo*-WE	0.137	0.131	0.118	0.220	
		*w*-WE	0.141	0.164	0.229	0.480	
	Mean values of the unnormalized |E| field						(V/m)
Oil-based cylindrical phantom		*wo*-WE	170.457	135.949	141.255	140.482	
		*w*-WE	152.245	189.240	150.249	180.131	
Human head model		*wo*-WE	141.255	140.482	131.268	252.349	
		*w*-WE	150.249	180.131	267.025	566.399	
	CV (SD/mean) of the unnormalized |$B_{1}^{+}$| field						(%)
Oil-based cylindrical phantom		*wo*-WE	1.688	1.4777	0.9695	0.3184	
		*w*-WE	1.086	2.8728	2.5087	2.6732	
Human head model		*wo*-WE	37.067	34.905	34.263	33.066	
		*w*-WE	35.753	34.325	33.789	33.363	
	CV (SD/mean) of the unnormalized |$B_{1}^{-}$| field						(%)
Oil-based cylindrical phantom		*wo*-WE	53.640	65.871	65.600	55.153	
		*w*-WE	60.450	65.036	65.840	64.886	
Human head model		*wo*-WE	35.139	37.433	39.082	40.073	
		*w*-WE	36.932	36.563	39.808	39.800	
	CV (SD/mean) of the unnormalized |E| field						(%)
Oil-based cylindrical phantom		*wo*-WE	35.306	32.687	109.103	367.758	
		*w*-WE	33.001	33.894	308.384	234.515	
Human head model		*wo*-WE	44.513	44.877	43.574	43.648	
		*w*-WE	44.175	44.320	43.325	43.452	

**Table 5:** Normalization coefficient (Norm-COEF).

		BP-BC RF Coil *wo*-*sc*HPM	BP-BC RF Coil *w*-*sc*HPM	16-ch RF Coil *wo*-*sc*HPM	16-ch RF Coil *w*-*sc*HPM	
Norm-COEF (Oil-based cylindrical phantom)						(a.u.)
*wo*-WE		0.793	0.723	0.264	0.085	
*w*-WE		0.689	0.521	0.076	0.071	
	Norm-COEF (Human head model)					(a.u.)
*wo*-WE		0.362	0.352	0.383	0.199	
*w*-WE		0.334	0.273	0.192	0.087	

**Table 6:** Mean values, SD values, and CV (SD/mean) in normalized |$B_{1}^{+}$| field simulation results using the oil-based cylindrical phantom and human head model.

			BP-BC RF Coil *wo*-*sc*HPM	BP-BC RF Coil *w*-*sc*HPM	16-ch RF Coil *wo*-*sc*HPM	16-ch RF Coil *w*-*sc*HPM	
	$\mathrm{Mean}\;\mathrm{values}\;\mathrm{of}\;\mathrm{the}\;\mathrm{normalized}\;|B_{1}^{+}|$ field						×10^−5^ (μT)
Oil-based cylindrical phantom		*wo*-WE	0.191	0.200	0.188	0.196	
		*w*-WE	0.196	0.203	0.190	0.200	
Human head model		*wo*-WE	0.096	0.100	0.091	0.095	
		*w*-WE	0.098	0.099	0.094	0.096	
	$\mathrm{SD}\;\mathrm{values}\;\mathrm{of}\;\mathrm{the}\;\mathrm{normalized}\;|B_{1}^{+}|$ field						×10^−5^ (μT)
Oil-based cylindrical phantom		*wo*-WE	0.002	0.005	0.003	0.003	
		*w*-WE	0.001	0.004	0.002	0.006	
Human head model		*wo*-WE	0.033	0.033	0.034	0.033	
		*w*-WE	0.033	0.033	0.034	0.033	
	$\mathrm{CV}\;(\mathrm{SD}/\mathrm{mean})\;\mathrm{of}\;\mathrm{the}\;\mathrm{normalized}\;|B_{1}^{+}|$ field						(%)
Oil-based cylindrical phantom		*wo*-WE	1.011	2.527	1.569	1.430	
		*w*-WE	0.409	2.183	1.022	2.902	
Human head model		*wo*-WE	34.304	33.084	37.142	35.043	
		*w*-WE	33.745	33.256	35.866	34.429	

**Table 7:** Whole-average and max SAR values of normalized EM field simulation results.

		BP-BC RF Coil *wo*-*sc*HPM	BP-BC RF Coil *w*-*sc*HPM	16-ch RF Coil *wo*-*sc*HPM	16-ch RF Coil *w*-*sc*HPM	
	Whole-averaged SAR (Mean SAR values)					(W/kg)
*wo*-WE		0.209	0.215	0.249	0.238	
*w*-WE		0.216	0.211	0.243	0.227	
	Max SAR values					(W/kg)
*wo*-WE		8.203	8.461	8.496	8.472	
*w*-WE		8.376	8.248	8.284	8.552	

## Data Availability

Not applicable.

## Supplementary Materials

The following supporting information can be downloaded at: https://www.mdpi.com/article/10.3390/s22228968/s1: Figure S1. Unnormalized |$B_{1}^{+}$| field distribution (*x*–*z* plane) in the oil-based cylindrical phantom (a–h) and human head model (i–p): (a,i) BP-BC RF coil – *wo*-*sc*HPM – *wo*-MCWE; (b,j) BP-BC RF coil – *w*-*sc*HPM – *wo*-MCWE; (c,k) 16-ch RF coil – *wo*-*sc*HPM – *wo*-BCWE; (d,j) 16-ch RF coil – *w*-*sc*HPM – *wo*-BCWE; (e,m) BP-BC RF coil – *wo*-*sc*HPM – *w*-MCWE; (f,l) BP-BC RF coil – *w*-*sc*HPM – *w*-MCWE; (g,o) 16-ch RF coil – *wo*-*sc*HPM – *w*-BCWE; (h,p) 16-ch RF coil – *w*-*sc*HPM – *w*-BCWE.; Figure S2. Unnormalized |$B_{1}^{+}$| field distribution (*y*–*z* plane) in the oil-based cylindrical phantom (a–h) and human head model (i–p): (a,i) BP-BC RF coil – *wo*-*sc*HPM – *wo*-MCWE; (b,j) BP-BC RF coil – *w*-*sc*HPM – *wo*-MCWE; (c,k) 16-ch RF coil – *wo*-*sc*HPM – *wo*-BCWE; (d,j) 16-ch RF coil – *w*-*sc*HPM – *wo*-BCWE; (e,m) BP-BC RF coil – *wo*-*sc*HPM – *w*-MCWE; (f,l) BP-BC RF coil – *w*-*sc*HPM – *w*-MCWE; (g,o) 16-ch RF coil – *wo*-*sc*HPM – *w*-BCWE; (h,p) 16-ch RF coil – *w*-*sc*HPM – *w*-BCWE.; Figure S3. Unnormalized |$B_{1}^{-}$| field distribution (*x*–*z* plane) in the oil-based cylindrical phantom (a–h) and human head model (i–p): (a,i) BP-BC RF coil – *wo*-*sc*HPM – *wo*-MCWE; (b,j) BP-BC RF coil – *w*-*sc*HPM – *wo*-MCWE; (c,k) 16-ch RF coil – *wo*-*sc*HPM – *wo*-BCWE; (d,j) 16-ch RF coil – *w*-*sc*HPM – *wo*-BCWE; (e,m) BP-BC RF coil – *wo*-*sc*HPM – *w*-MCWE; (f,l) BP-BC RF coil – *w*-*sc*HPM – *w*-MCWE; (g,o) 16-ch RF coil – *wo*-*sc*HPM – *w*-BCWE; (h,p) 16-ch RF coil – *w*-*sc*HPM – *w*-BCWE.; Figure S4. Unnormalized |$B_{1}^{-}$| field distribution (*y*–*z* plane) in the oil-based cylindrical phantom (a–h) and human head model (i–p): (a,i) BP-BC RF coil – *wo*-*sc*HPM – *wo*-MCWE; (b,j) BP-BC RF coil – *w*-*sc*HPM – *wo*-MCWE; (c,k) 16-ch RF coil – *wo*-*sc*HPM – *wo*-BCWE; (d,j) 16-ch RF coil – *w*-*sc*HPM – *wo*-BCWE; (e,m) BP-BC RF coil – *wo*-*sc*HPM – *w*-MCWE; (f,l) BP-BC RF coil – *w*-*sc*HPM – *w*-MCWE; (g,o) 16-ch RF coil – *wo*-*sc*HPM – *w*-BCWE; (h,p) 16-ch RF coil – *w*-*sc*HPM – *w*-BCWE.; Figure S5. Unnormalized |E| field distribution (*x*–*z* plane) in the oil-based cylindrical phantom (a–h) and human head model (i–p): (a,i) BP-BC RF coil – *wo*-*sc*HPM – *wo*-MCWE; (b,j) BP-BC RF coil – *w*-*sc*HPM – *wo*-MCWE; (c,k) 16-ch RF coil – *wo*-*sc*HPM – *wo*-BCWE; (d,j) 16-ch RF coil – *w*-*sc*HPM – *wo*-BCWE; (e,m) BP-BC RF coil – *wo*-*sc*HPM – *w*-MCWE; (f,l) BP-BC RF coil – *w*-*sc*HPM – *w*-MCWE; (g,o) 16-ch RF coil – *wo*-*sc*HPM – *w*-BCWE; (h,p) 16-ch RF coil – *w*-*sc*HPM – *w*-BCWE.; Figure S6. Unnormalized |E| field distribution (*y*–*z* plane) in the oil-based cylindrical phantom (a–h) and human head model (i–p): (a,i) BP-BC RF coil – *wo*-*sc*HPM – *wo*-MCWE; (b,j) BP-BC RF coil – *w*-*sc*HPM – *wo*-MCWE; (c,k) 16-ch RF coil – *wo*-*sc*HPM – *wo*-BCWE; (d,j) 16-ch RF coil – *w*-*sc*HPM – *wo*-BCWE; (e,m) BP-BC RF coil – *wo*-*sc*HPM – *w*-MCWE; (f,l) BP-BC RF coil – *w*-*sc*HPM – *w*-MCWE; (g,o) 16-ch RF coil – *wo*-*sc*HPM – *w*-BCWE; (h,p) 16-ch RF coil – *w*-*sc*HPM – *w*-BCWE.; Figure S7. Normalized |$B_{1}^{+}$| field distribution (*x*–*z* plane) in the oil-based cylindrical phantom (a–h) and human head model (i–p): (a,i) BP-BC RF coil – *wo*-*sc*HPM – *wo*-MCWE; (b,j) BP-BC RF coil – *w*-*sc*HPM – *wo*-MCWE; (c,k) 16-ch RF coil – *wo*-*sc*HPM – *wo*-BCWE; (d,j) 16-ch RF coil – *w*-*sc*HPM – *wo*-BCWE; (e,m) BP-BC RF coil – *wo*-*sc*HPM – *w*-MCWE; (f,l) BP-BC RF coil – *w*-*sc*HPM – *w*-MCWE; (g,o) 16-ch RF coil – *wo*-*sc*HPM – *w*-BCWE; (h,p) 16-ch RF coil – *w*-*sc*HPM – *w*-BCWE.; Figure S8. Normalized |$B_{1}^{+}$| field distribution (*y*–*z* plane) in the oil-based cylindrical phantom (a–h) and human head model (i–p): (a,i) BP-BC RF coil – *wo*-*sc*HPM – *wo*-MCWE; (b,j) BP-BC RF coil – *w*-*sc*HPM – *wo*-MCWE; (c,k) 16-ch RF coil – *wo*-*sc*HPM – *wo*-BCWE; (d,j) 16-ch RF coil – *w*-*sc*HPM – *wo*-BCWE; (e,m) BP-BC RF coil – *wo*-*sc*HPM – *w*-MCWE; (f,l) BP-BC RF coil – *w*-*sc*HPM – *w*-MCWE; (g,o) 16-ch RF coil – *wo*-*sc*HPM – *w*-BCWE; (h,p) 16-ch RF coil – *w*-*sc*HPM – *w*-BCWE.; Figure S9. Normalized SAR maps (*x*–*z* plane) constructed using Norm-COEF values: (a) BP-BC RF coil – *wo*-*sc*HPM – *wo*-MCWE; (b) BP-BC RF coil – *w*-*sc*HPM – *wo*-MCWE; (c) 16-ch RF coil – *wo*-*sc*HPM – *wo*-BCWE; (d) 16-ch RF coil – *w*-*sc*HPM – *wo*-BCWE; (e) BP-BC RF coil – *wo*-*sc*HPM – *w*-MCWE; (f) BP-BC RF coil – *w*-*sc*HPM – *w*-MCWE; (g) 16-ch RF coil – *wo*-*sc*HPM – *w*-BCWE; (h) 16-ch RF coil – *w*-*sc*HPM – *w*-BCWE.; Figure S10. Normalized SAR maps (*y*–*z* plane) constructed using Norm-COEF values: (a) BP-BC RF coil – *wo*-*sc*HPM – *wo*-MCWE; (b) BP-BC RF coil – *w*-*sc*HPM – *wo*-MCWE; (c) 16-ch RF coil – *wo*-*sc*HPM – *wo*-BCWE; (d) 16-ch RF coil – *w*-*sc*HPM – *wo*-BCWE; (e) BP-BC RF coil – *wo*-*sc*HPM – *w*-MCWE; (f) BP-BC RF coil – *w*-*sc*HPM – *w*-MCWE; (g) 16-ch RF coil – *wo*-*sc*HPM – *w*-BCWE; (h) 16-ch RF coil – *w*-*sc*HPM – *w*-BCWE. Figure S11. Unnormalized |$B_{1}$| field distribution (*x*–*y* plane) in the oil-based cylindrical phantom (a–h) and human head model (i–p): (a,i) BP-BC RF coil – *wo*-*sc*HPM – *wo*-MCWE; (b,j) BP-BC RF coil – *w*-*sc*HPM – *wo*-MCWE; (c,k) 16-ch RF coil – *wo*-*sc*HPM – *wo*-BCWE; (d,j) 16-ch RF coil – *w*-*sc*HPM – *wo*-BCWE; (e,m) BP-BC RF coil – *wo*-*sc*HPM – *w*-MCWE; (f,l) BP-BC RF coil – *w*-*sc*HPM – *w*-MCWE; (g,o) 16-ch RF coil – *wo*-*sc*HPM – *w*-BCWE; (h,p) 16-ch RF coil – *w*-*sc*HPM – *w*-BCWE.; Figure S12. Unnormalized |$B_{1}$| field distribution (*x*–*z* plane) in the oil-based cylindrical phantom (a–h) and human head model (i–p): (a,i) BP-BC RF coil – *wo*-*sc*HPM – *wo*-MCWE; (b,j) BP-BC RF coil – *w*-*sc*HPM – *wo*-MCWE; (c,k) 16-ch RF coil – *wo*-*sc*HPM – *wo*-BCWE; (d,j) 16-ch RF coil – *w*-*sc*HPM – *wo*-BCWE; (e,m) BP-BC RF coil – *wo*-*sc*HPM – *w*-MCWE; (f,l) BP-BC RF coil – *w*-*sc*HPM – *w*-MCWE; (g,o) 16-ch RF coil – *wo*-*sc*HPM – *w*-BCWE; (h,p) 16-ch RF coil – *w*-*sc*HPM – *w*-BCWE.; Figure S13. Unnormalized |$B_{1}$| field distribution (*y*–*z* plane) in the oil-based cylindrical phantom (a–h) and human head model (i–p): (a,i) BP-BC RF coil – *wo*-*sc*HPM – *wo*-MCWE; (b,j) BP-BC RF coil – *w*-*sc*HPM – *wo*-MCWE; (c,k) 16-ch RF coil – *wo*-*sc*HPM – *wo*-BCWE; (d,j) 16-ch RF coil – *w*-*sc*HPM – *wo*-BCWE; (e,m) BP-BC RF coil – *wo*-*sc*HPM – *w*-MCWE; (f,l) BP-BC RF coil – *w*-*sc*HPM – *w*-MCWE; (g,o) 16-ch RF coil – *wo*-*sc*HPM – *w*-BCWE; (h,p) 16-ch RF coil – *w*-*sc*HPM – *w*-BCWE.
