# Supplementary material for: A New Combination of Radio-Frequency Coil Configurations Using High-Permittivity Materials and Inductively Coupled Structures for Ultrahigh-Field Magnetic Resonance Imaging
Source: Sensors (Basel). 2022 Nov 19;22(22):8968. doi: 10.3390/s22228968 (PMC9694602; doi:10.3390/s22228968)
Supplement: Supplementary file 1 [file sensors-22-08968-s001.zip › sensors-1912232-supplementary.pdf]

sensors

Supplementary Materials

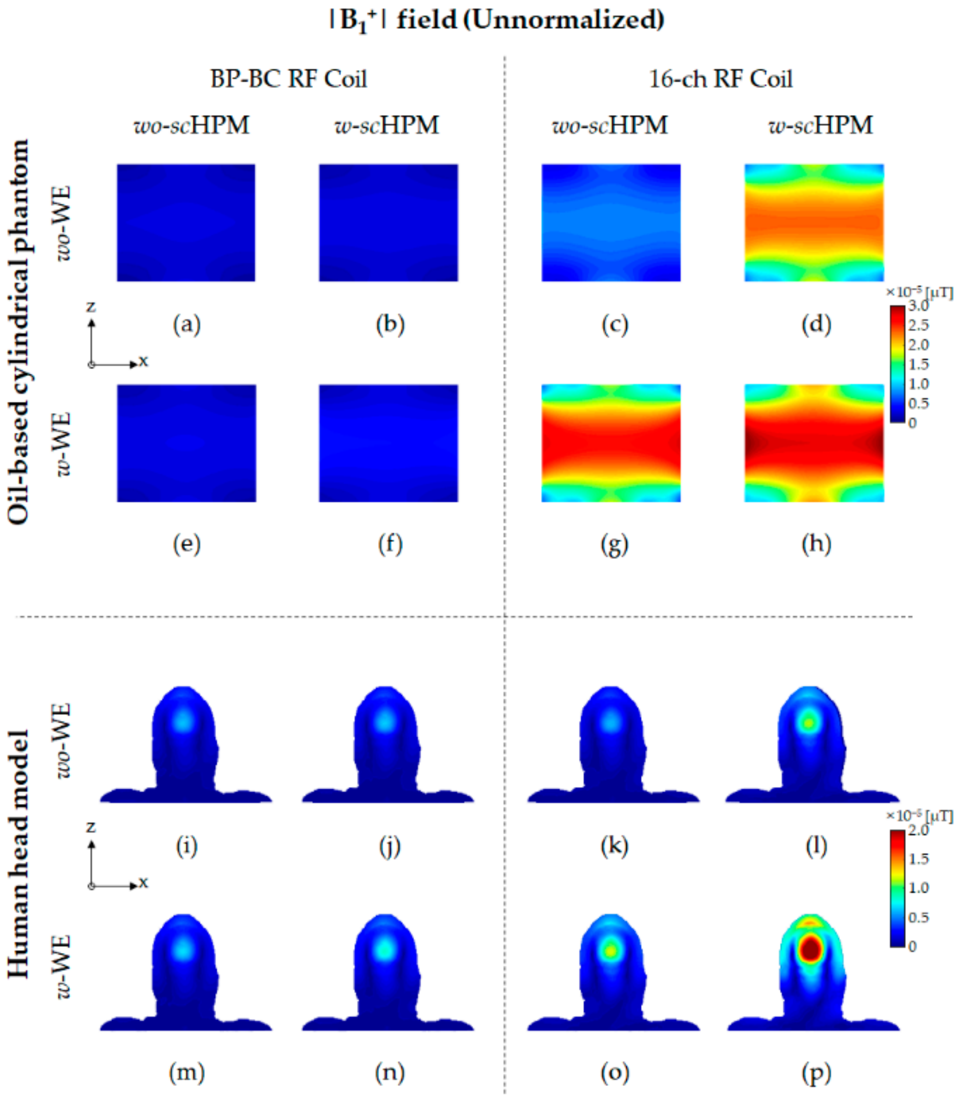

Figure S1. Unnormalized $|B_1^+|$ field distribution (*x*–*z* plane) in the oil-based cylindrical phantom (**a**–**h**) and human head model (**i**–**p**): (**a**,**i**) BP-BC RF coil – *wo*-*sc*HPM – *wo*-MCWE; (**b**,**j**) BP-BC RF coil – *w*-*sc*HPM – *wo*-MCWE; (**c**,**k**) 16-ch RF coil – *wo*-*sc*HPM – *wo*-BCWE; (**d**,**j**) 16-ch RF coil – *w*-*sc*HPM – *wo*-BCWE; (**e**,**m**) BP-BC RF coil – *wo*-*sc*HPM – *w*-MCWE; (**f**,**l**) BP-BC RF coil – *w*-*sc*HPM – *w*-MCWE; (**g**,**o**) 16-ch RF coil – *wo*-*sc*HPM – *w*-BCWE; (**h**,**p**) 16-ch RF coil – *w*-*sc*HPM – *w*-BCWE.

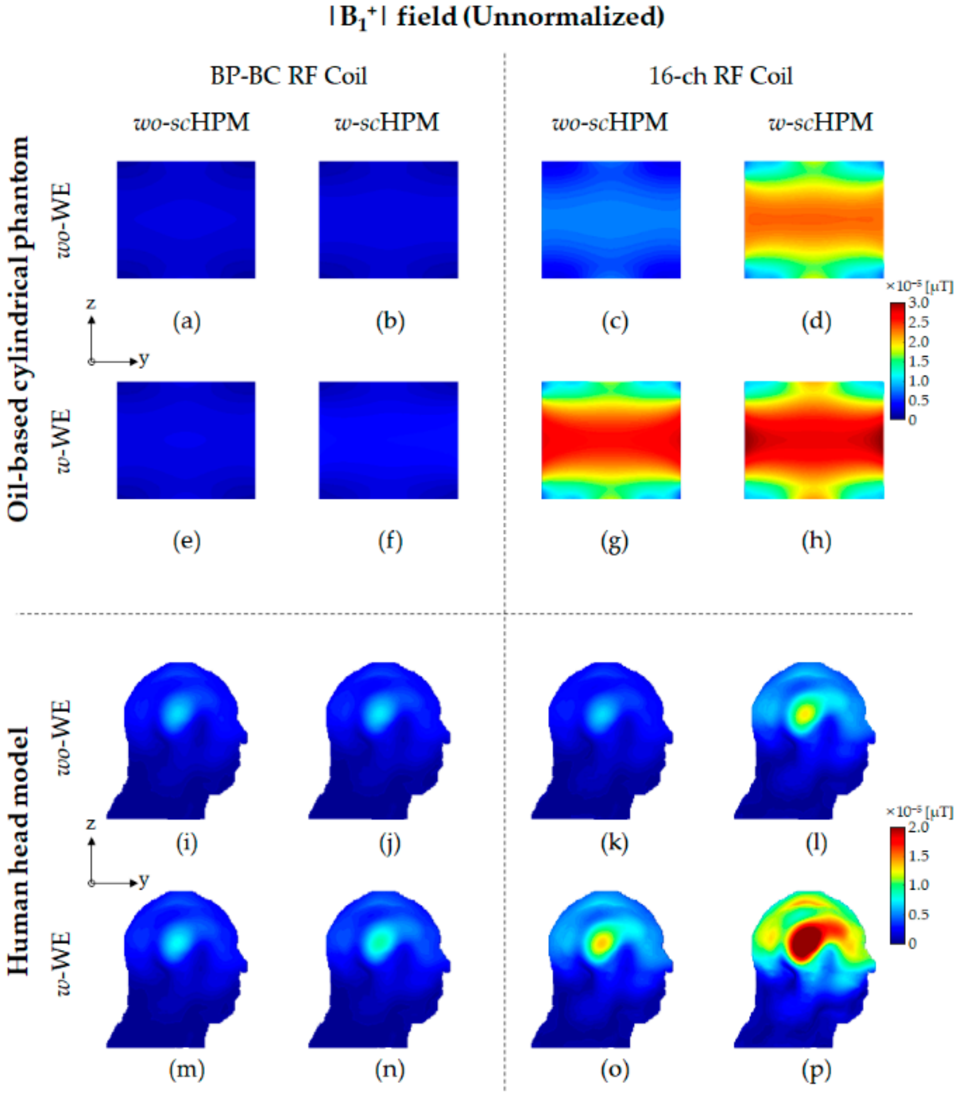

Figure S2. Unnormalized $|B_1^+|$ field distribution (*y*–*z* plane) in the oil-based cylindrical phantom (**a**–**h**) and human head model (**i**–**p**): (**a**,**i**) BP-BC RF coil – *wo*-*sc*HPM – *wo*-MCWE; (**b**,**j**) BP-BC RF coil – *w*-*sc*HPM – *wo*-MCWE; (**c**,**k**) 16-ch RF coil – *wo*-*sc*HPM – *wo*-BCWE; (**d**,**j**) 16-ch RF coil – *w*-*sc*HPM – *wo*-BCWE; (**e**,**m**) BP-BC RF coil – *wo*-*sc*HPM – *w*-MCWE; (**f**,**l**) BP-BC RF coil – *w*-*sc*HPM – *w*-MCWE; (**g**,**o**) 16-ch RF coil – *wo*-*sc*HPM – *w*-BCWE; (**h**,**p**) 16-ch RF coil – *w*-*sc*HPM – *w*-BCWE.

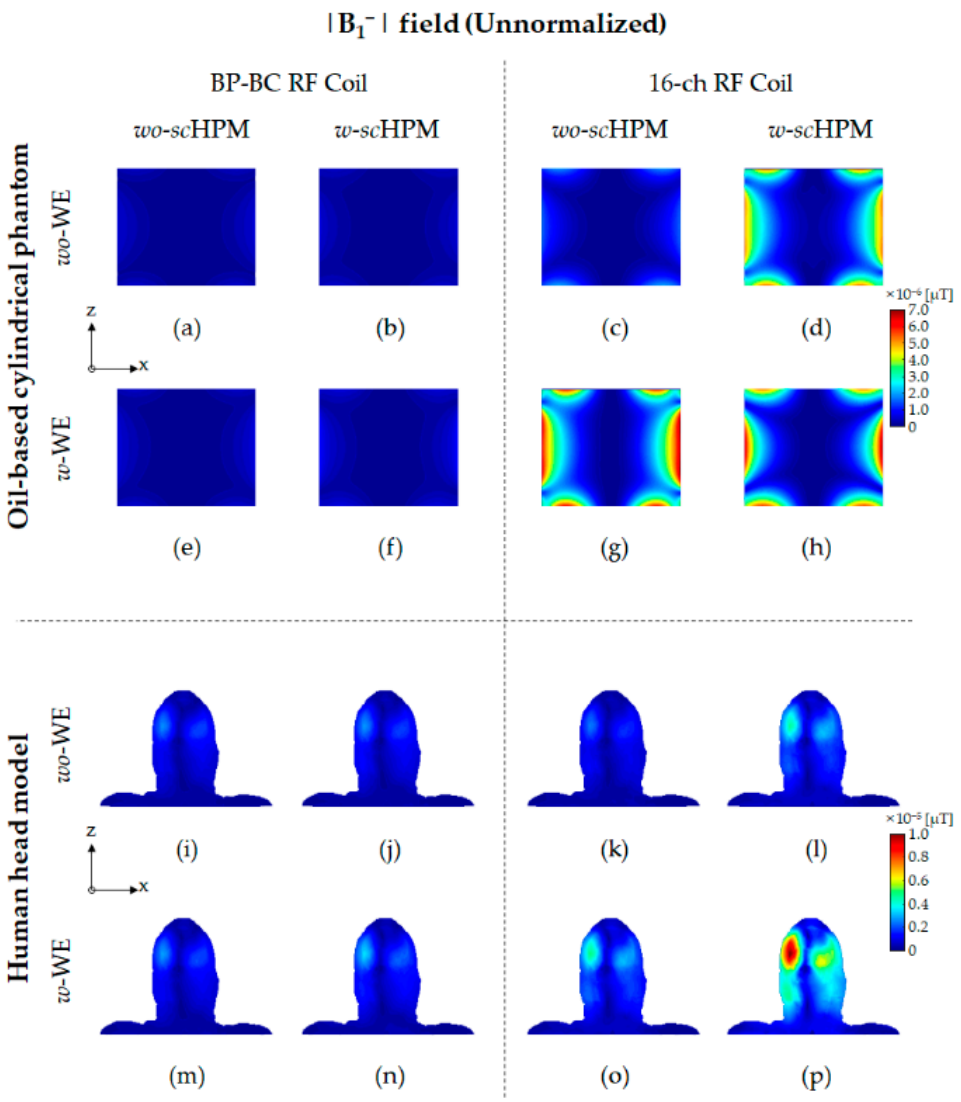

Figure S3. Unnormalized $|B_1^-|$ field distribution (*x*–*z* plane) in the oil-based cylindrical phantom (**a**–**h**) and human head model (**i**–**p**): (**a**,**i**) BP-BC RF coil – *wo*-*sc*HPM – *wo*-MCWE; (**b**,**j**) BP-BC RF coil – *w*-*sc*HPM – *wo*-MCWE; (**c**,**k**) 16-ch RF coil – *wo*-*sc*HPM – *wo*-BCWE; (**d**,**j**) 16-ch RF coil – *w*-*sc*HPM – *wo*-BCWE; (**e**,**m**) BP-BC RF coil – *wo*-*sc*HPM – *w*-MCWE; (**f**,**l**) BP-BC RF coil – *w*-*sc*HPM – *w*-MCWE; (**g**,**o**) 16-ch RF coil – *wo*-*sc*HPM – *w*-BCWE; (**h**,**p**) 16-ch RF coil – *w*-*sc*HPM – *w*-BCWE.

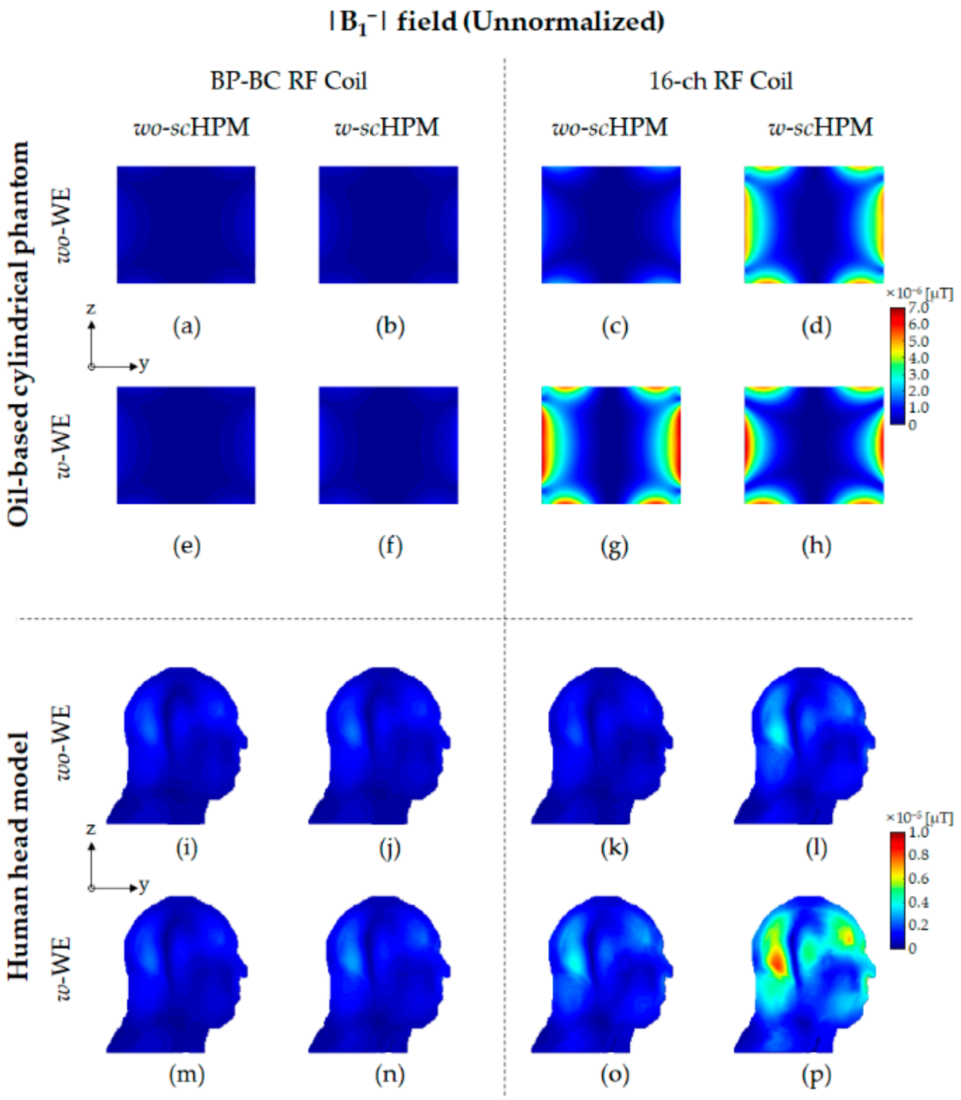

Figure S4. Unnormalized $|B_1^-|$ field distribution (*y*–*z* plane) in the oil-based cylindrical phantom (**a**–**h**) and human head model (**i**–**p**): (**a**,**i**) BP-BC RF coil – *wo*-*sc*HPM – *wo*-MCWE; (**b**,**j**) BP-BC RF coil – *w*-*sc*HPM – *wo*-MCWE; (**c**,**k**) 16-ch RF coil – *wo*-*sc*HPM – *wo*-BCWE; (**d**,**j**) 16-ch RF coil – *w*-*sc*HPM – *wo*-BCWE; (**e**,**m**) BP-BC RF coil – *wo*-*sc*HPM – *w*-MCWE; (**f**,**l**) BP-BC RF coil – *w*-*sc*HPM – *w*-MCWE; (**g**,**o**) 16-ch RF coil – *wo*-*sc*HPM – *w*-BCWE; (**h**,**p**) 16-ch RF coil – *w*-*sc*HPM – *w*-BCWE.

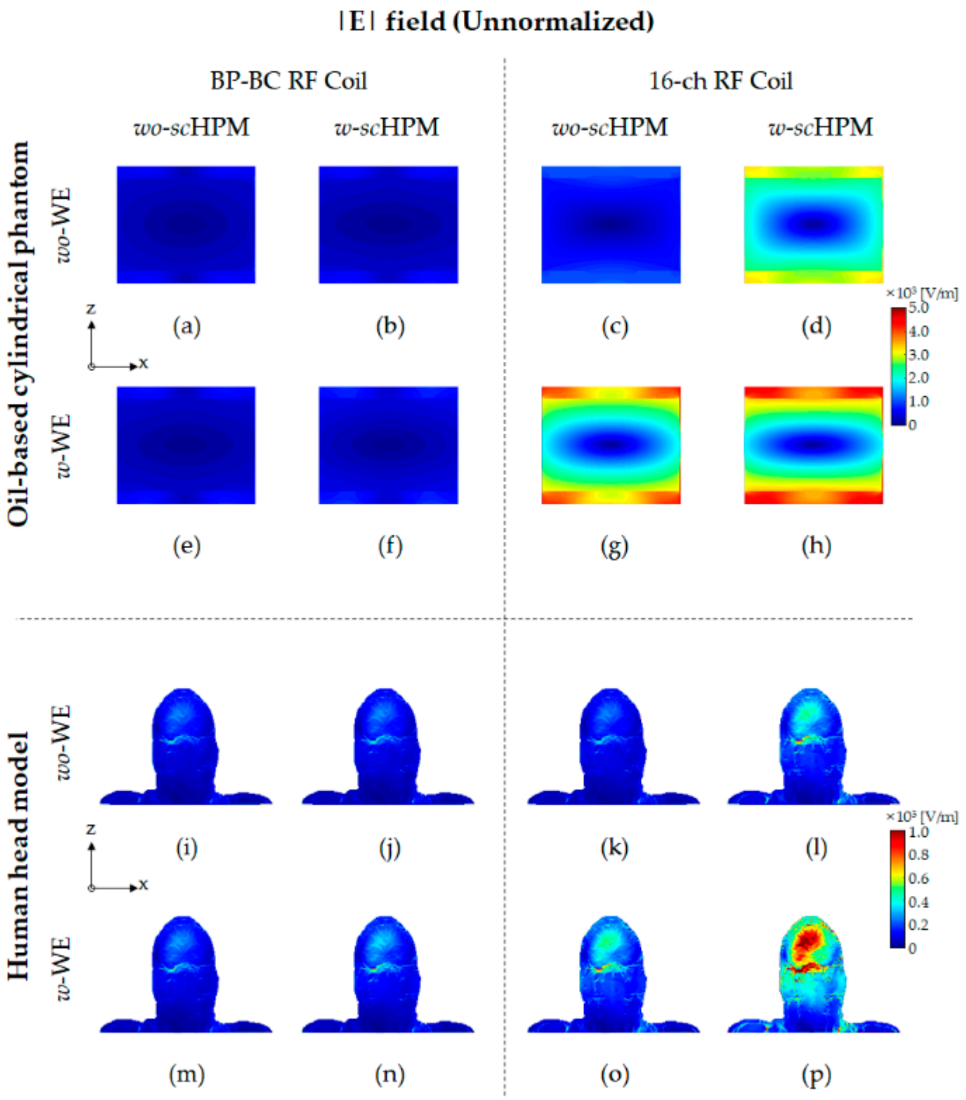

Figure S5. Unnormalized |E| field distribution (*x*–*z* plane) in the oil-based cylindrical phantom (**a**–**h**) and human head model (**i**–**p**): (**a**,**i**) BP-BC RF coil – *wo*-*sc*HPM – *wo*-MCWE; (**b**,**j**) BP-BC RF coil – *w*-*sc*HPM – *wo*-MCWE; (**c**,**k**) 16-ch RF coil – *wo*-*sc*HPM – *wo*-BCWE; (**d**,**j**) 16-ch RF coil – *w*-*sc*HPM – *wo*-BCWE; (**e**,**m**) BP-BC RF coil – *wo*-*sc*HPM – *w*-MCWE; (**f**,**l**) BP-BC RF coil – *w*-*sc*HPM – *w*-MCWE; (**g**,**o**) 16-ch RF coil – *wo*-*sc*HPM – *w*-BCWE; (**h**,**p**) 16-ch RF coil – *w*-*sc*HPM – *w*-BCWE.

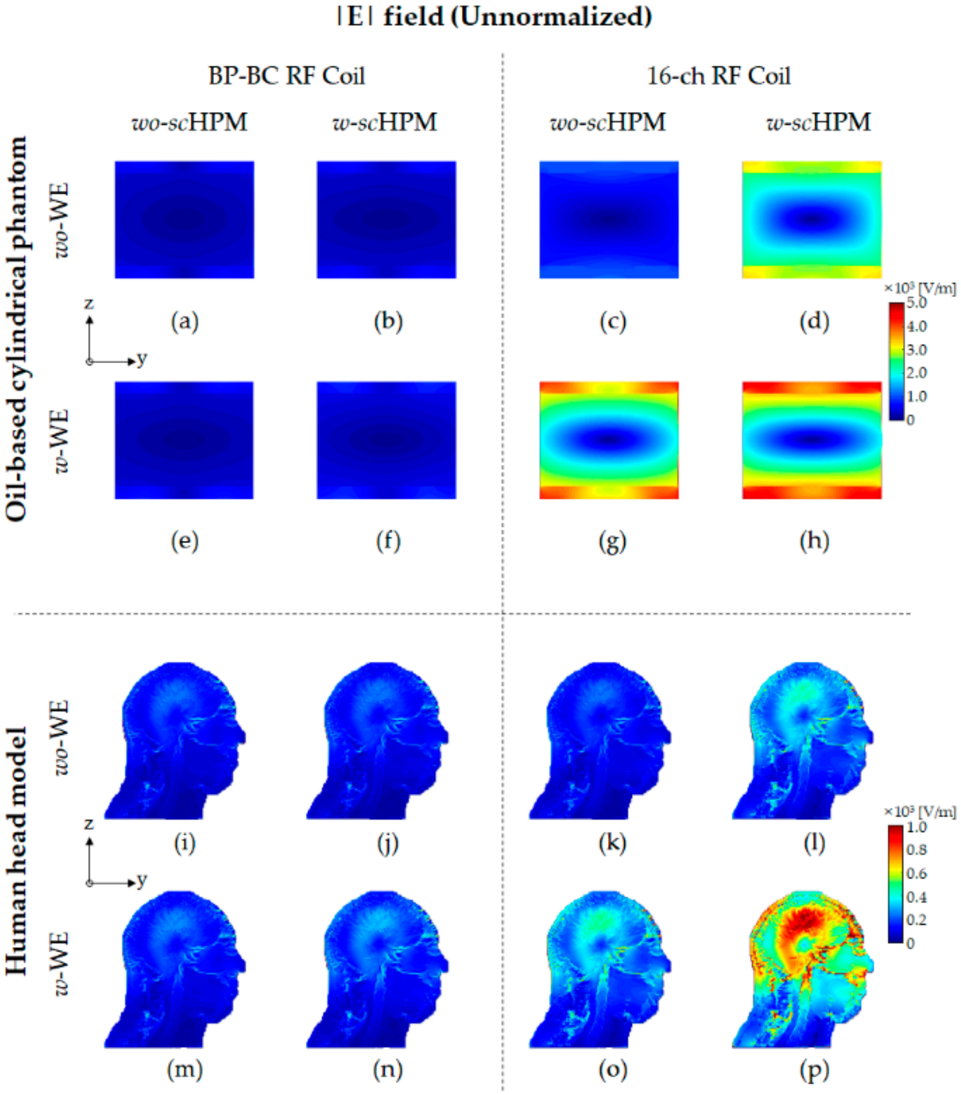

Figure S6. Unnormalized |E| field distribution (*y*–*z* plane) in the oil-based cylindrical phantom (**a**–**h**) and human head model (**i**–**p**): (**a**,**i**) BP-BC RF coil – *wo*-*sc*HPM – *wo*-MCWE; (**b**,**j**) BP-BC RF coil – *w*-*sc*HPM – *wo*-MCWE; (**c**,**k**) 16-ch RF coil – *wo*-*sc*HPM – *wo*-BCWE; (**d**,**j**) 16-ch RF coil – *w*-*sc*HPM – *wo*-BCWE; (**e**,**m**) BP-BC RF coil – *wo*-*sc*HPM – *w*-MCWE; (**f**,**l**) BP-BC RF coil – *w*-*sc*HPM – *w*-MCWE; (**g**,**o**) 16-ch RF coil – *wo*-*sc*HPM – *w*-BCWE; (**h**,**p**) 16-ch RF coil – *w*-*sc*HPM – *w*-BCWE.

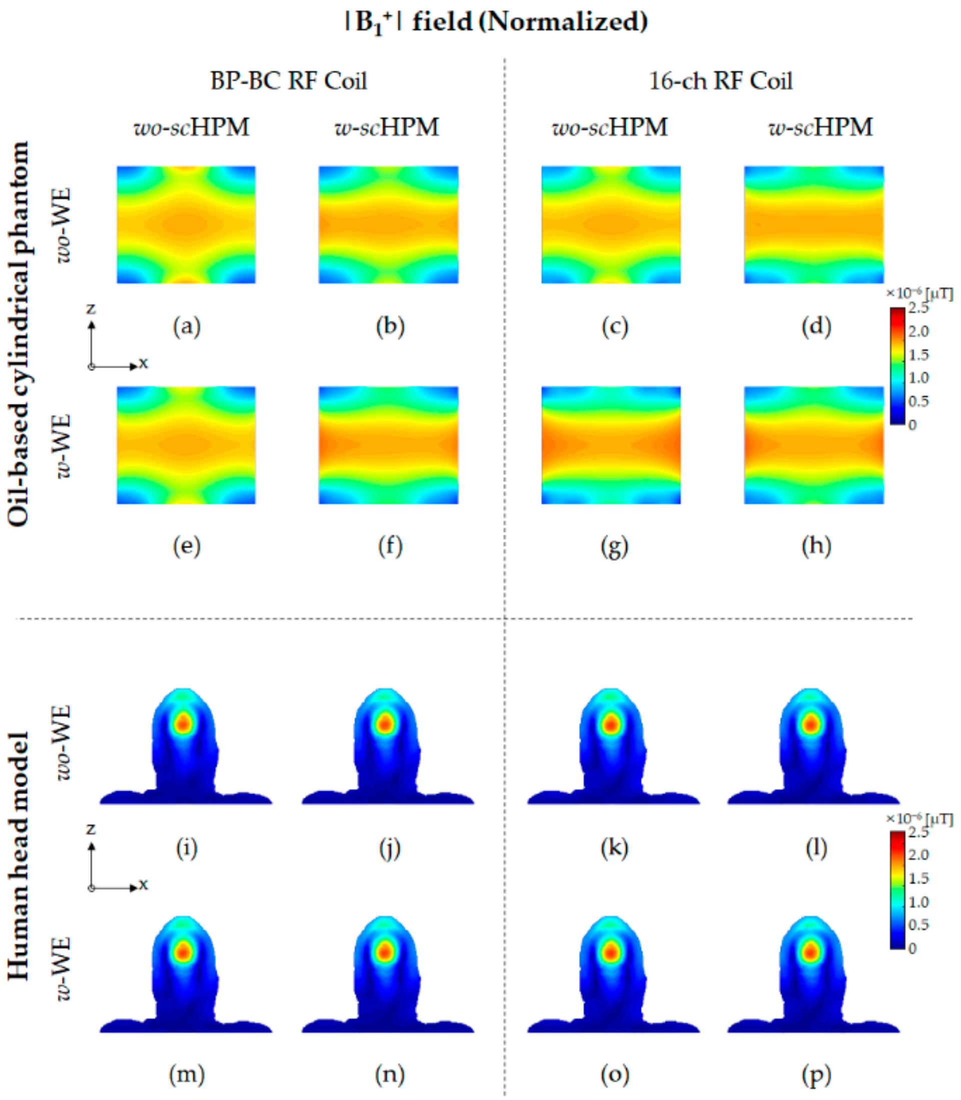

Figure S7. Normalized $|B_1^+|$ field distribution (x–z plane) in the oil-based cylindrical phantom (**a**–**h**) and human head model (**i**–**p**): (**a**,**i**) BP-BC RF coil – *wo-sc*HPM – *wo*-MCWE; (**b**,**j**) BP-BC RF coil – *w-sc*HPM – *wo*-MCWE; (**c**,**k**) 16-ch RF coil – *wo-sc*HPM – *wo*-BCWE; (**d**,**j**) 16-ch RF coil – *w-sc*HPM – *wo*-BCWE; (**e**,**m**) BP-BC RF coil – *wo-sc*HPM – *w*-MCWE; (**f**,**l**) BP-BC RF coil – *w-sc*HPM – *w*-MCWE; (**g**,**o**) 16-ch RF coil – *wo-sc*HPM – *w*-BCWE; (**h**,**p**) 16-ch RF coil – *w-sc*HPM – *w*-BCWE.

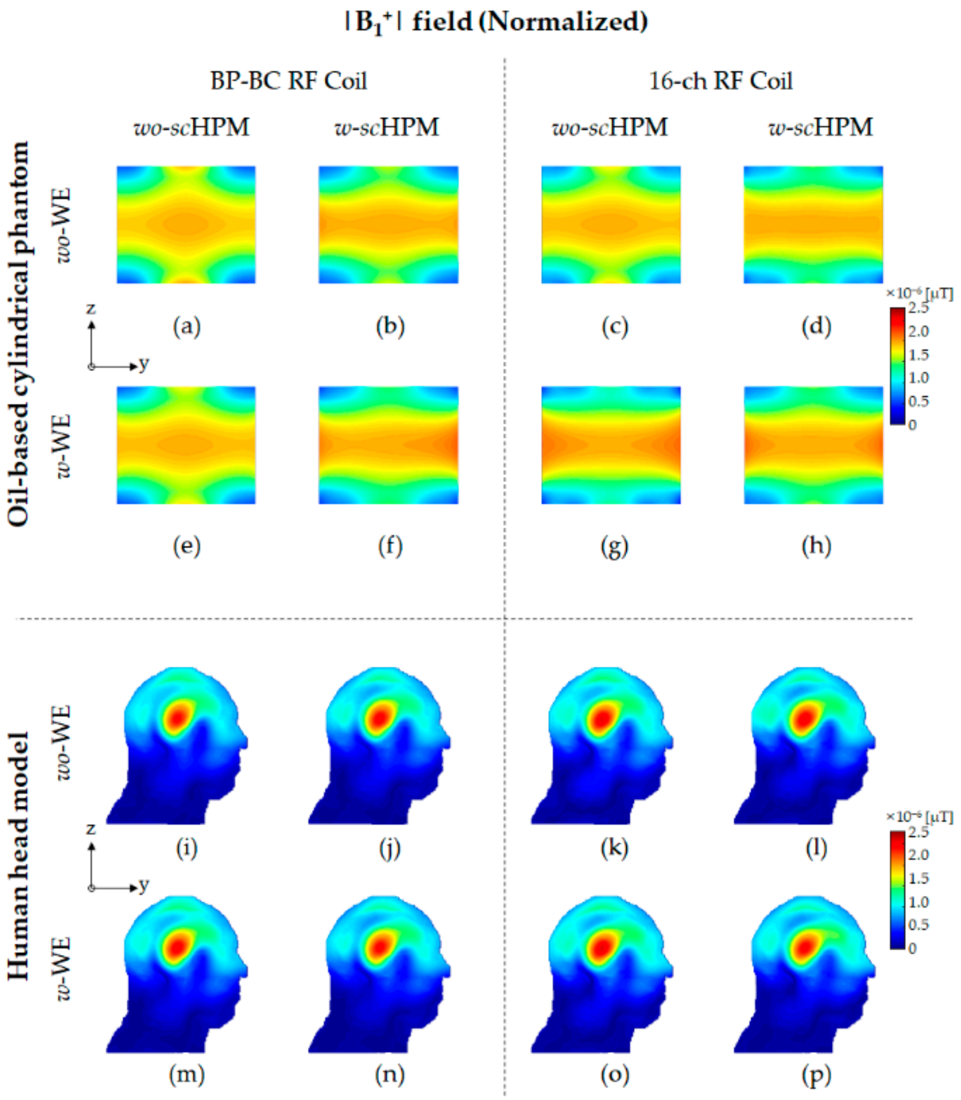

Figure S8. Normalized $|B_1^+|$ field distribution (*y*–*z* plane) in the oil-based cylindrical phantom (**a**–**h**) and human head model (**i**–**p**): (**a**,**i**) BP-BC RF coil – *wo-sc*HPM – *wo*-MCWE; (**b**,**j**) BP-BC RF coil – *w-sc*HPM – *wo*-MCWE; (**c**,**k**) 16-ch RF coil – *wo-sc*HPM – *wo*-BCWE; (**d**,**j**) 16-ch RF coil – *w-sc*HPM – *wo*-BCWE; (**e**,**m**) BP-BC RF coil – *wo-sc*HPM – *w*-MCWE; (**f**,**l**) BP-BC RF coil – *w-sc*HPM – *w*-MCWE; (**g**,**o**) 16-ch RF coil – *wo-sc*HPM – *w*-BCWE; (**h**,**p**) 16-ch RF coil – *w-sc*HPM – *w*-BCWE.

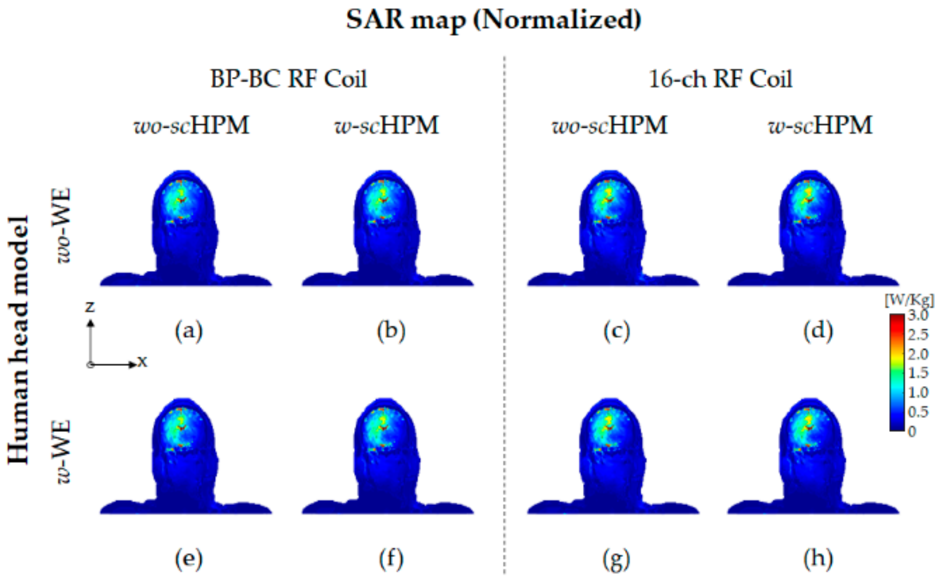

Figure S9. Normalized SAR maps (x–z plane) in the oil-based cylindrical phantom (**a**–**h**) and human head model (**i**–**p**): (**a**,**i**) BP-BC RF coil – *wo*-*sc*HPM – *wo*-MCWE; (**b**,**j**) BP-BC RF coil – *w*-*sc*HPM – *wo*-MCWE; (**c**,**k**) 16-ch RF coil – *wo*-*sc*HPM – *wo*-BCWE; (**d**,**j**) 16-ch RF coil – *w*-*sc*HPM – *wo*-BCWE; (**e**,**m**) BP-BC RF coil – *wo*-*sc*HPM – *w*-MCWE; (**f**,**l**) BP-BC RF coil – *w*-*sc*HPM – *w*-MCWE; (**g**,**o**) 16-ch RF coil – *wo*-*sc*HPM – *w*-BCWE; (**h**,**p**) 16-ch RF coil – *w*-*sc*HPM – *w*-BCWE.

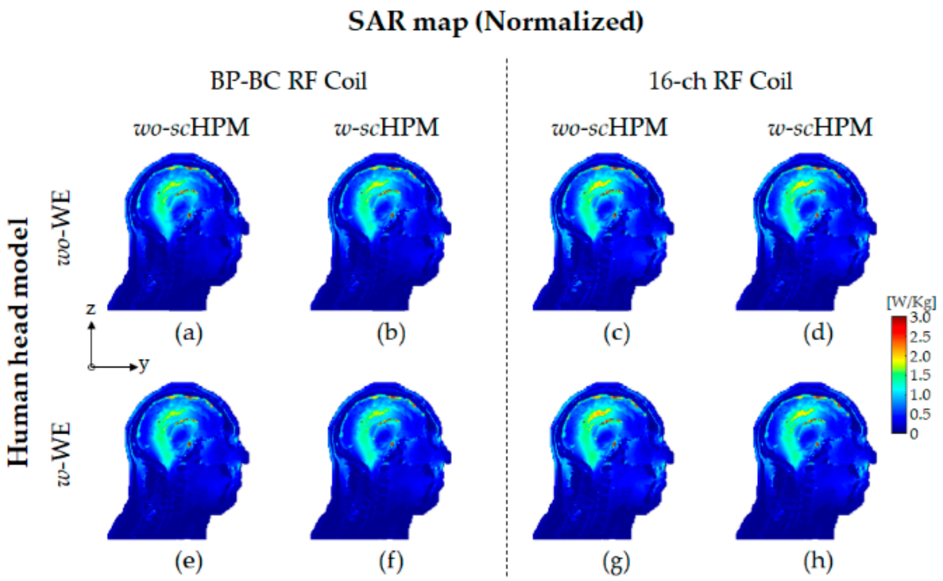

Figure S10. Normalized SAR maps (*y*–*z* plane) in the oil-based cylindrical phantom (**a**–**h**) and human head model (**i**–**p**): (**a**,**i**) BP-BC RF coil – *wo-sc*HPM – *wo*-MCWE; (**b**,**j**) BP-BC RF coil – *w-sc*HPM – *wo*-MCWE; (**c**,**k**) 16-ch RF coil – *wo-sc*HPM – *wo*-BCWE; (**d**,**j**) 16-ch RF coil – *w-sc*HPM – *wo*-BCWE; (**e**,**m**) BP-BC RF coil – *wo-sc*HPM – *w*-MCWE; (**f**,**l**) BP-BC RF coil – *w-sc*HPM – *w*-MCWE; (**g**,**o**) 16-ch RF coil – *wo-sc*HPM – *w*-BCWE; (**h**,**p**) 16-ch RF coil – *w-sc*HPM – *w*-BCWE.

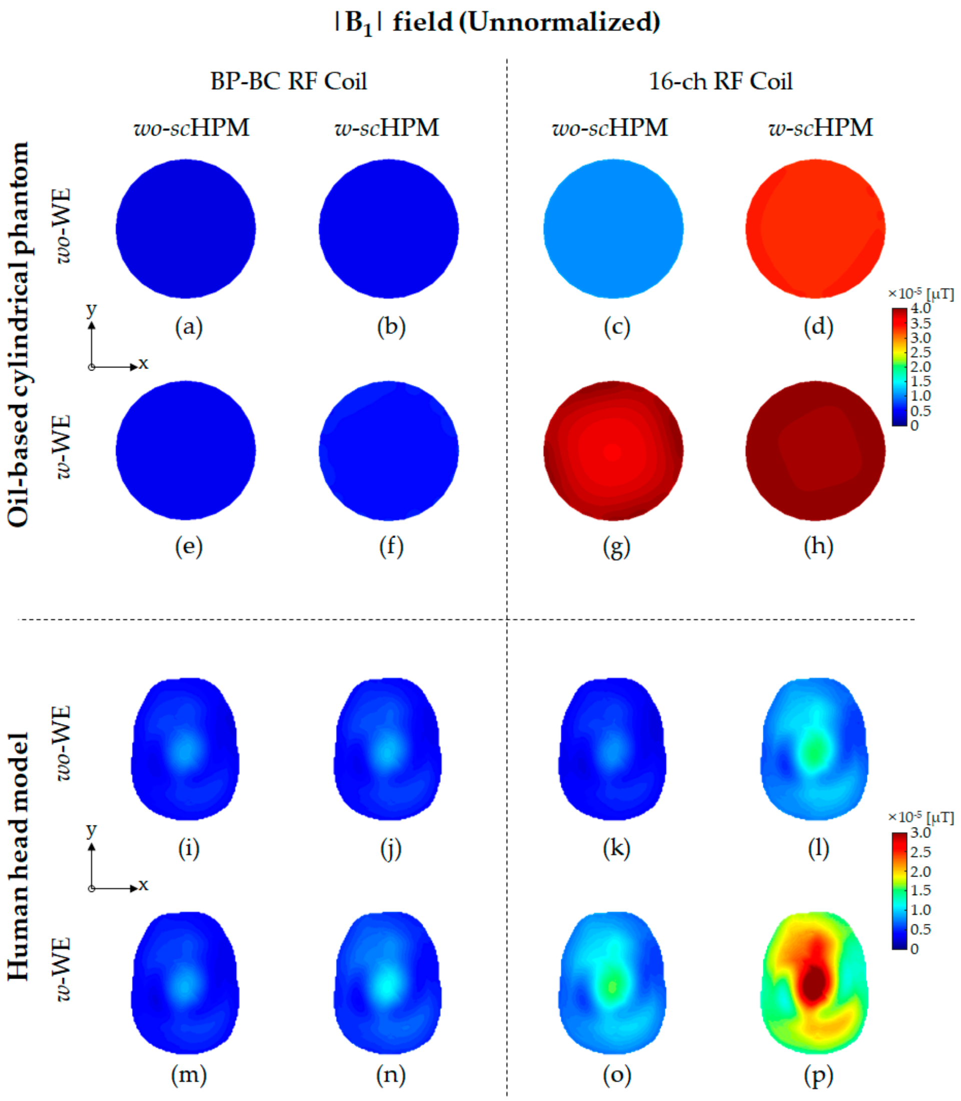

Figure S11. Unnormalized $|B_1|$ field distributions using the oil-based cylindrical phantom (**a**–**h**) and human head model (**i**–**p**): (**a**,i) BP-BC RF coil – *wo*-*sc*HPM – *wo*-MCWE; (**b**,j) BP-BC RF coil – *w*-*sc*HPM – *wo*-MCWE; (**c**,**k**) 16-ch RF coil – *wo*-*sc*HPM – *wo*-BCWE; (**d**,**j**) 16-ch RF coil – *w*-*sc*HPM – *wo*-BCWE; (**e**,**m**) BP-BC RF coil – *wo*-*sc*HPM – *w*-MCWE; (**f**,**l**) BP-BC RF coil – *w*-*sc*HPM – *w*-MCWE; (**g**,**o**) 16-ch RF coil – *wo*-*sc*HPM – *w*-BCWE; (**h**,**p**) 16-ch RF coil – *w*-*sc*HPM – *w*-BCWE.

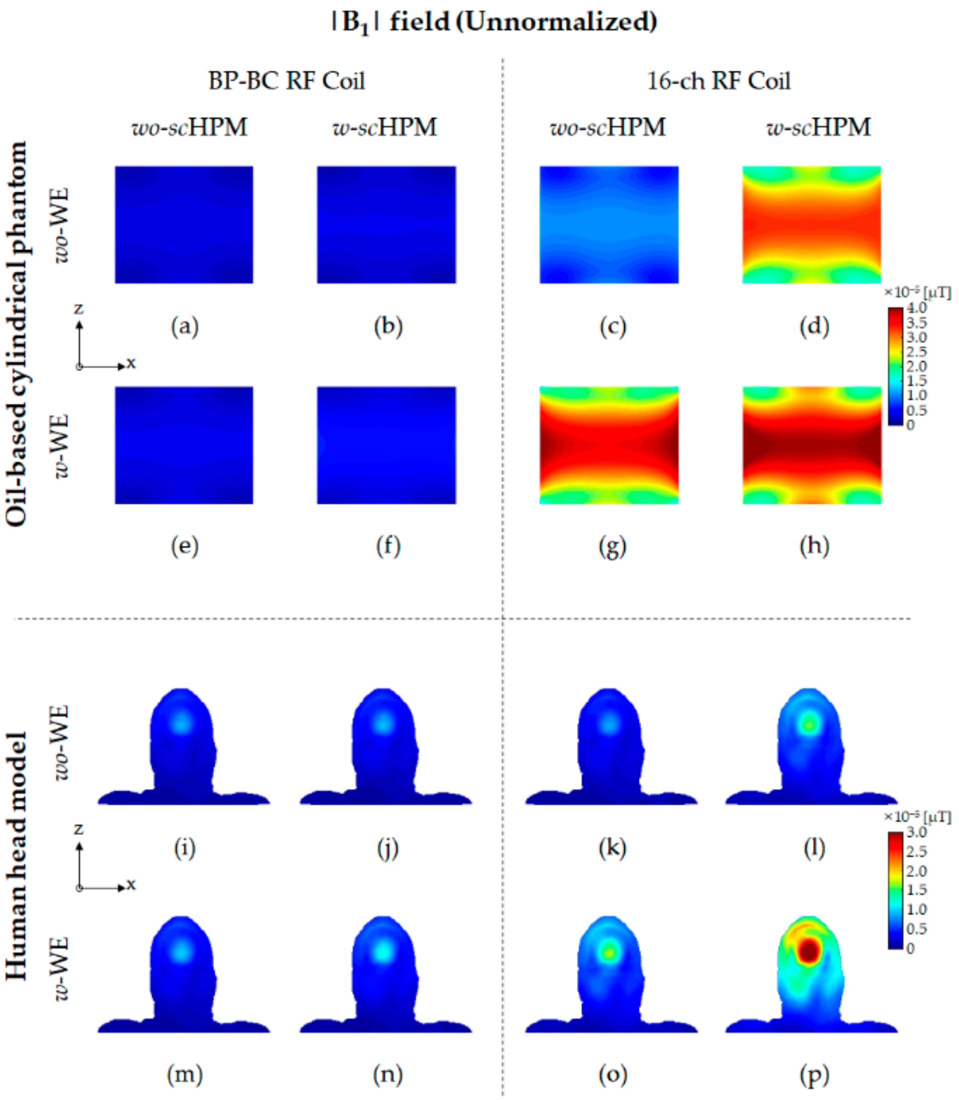

Figure S12. Unnormalized $|B_1|$ field distribution (*x*–*z* plane) in the oil-based cylindrical phantom (**a**–**h**) and human head model (**i**–**p**): (**a**,**i**) BP-BC RF coil – *wo*-*sc*HPM – *wo*-MCWE; (**b**,**j**) BP-BC RF coil – *w*-*sc*HPM – *wo*-MCWE; (**c**,**k**) 16-ch RF coil – *wo*-*sc*HPM – *wo*-BCWE; (**d**,**j**) 16-ch RF coil – *w*-*sc*HPM – *wo*-BCWE; (**e**,**m**) BP-BC RF coil – *wo*-*sc*HPM – *w*-MCWE; (**f**,**l**) BP-BC RF coil – *w*-*sc*HPM – *w*-MCWE; (**g**,**o**) 16-ch RF coil – *wo*-*sc*HPM – *w*-BCWE; (**h**,**p**) 16-ch RF coil – *w*-*sc*HPM – *w*-BCWE.

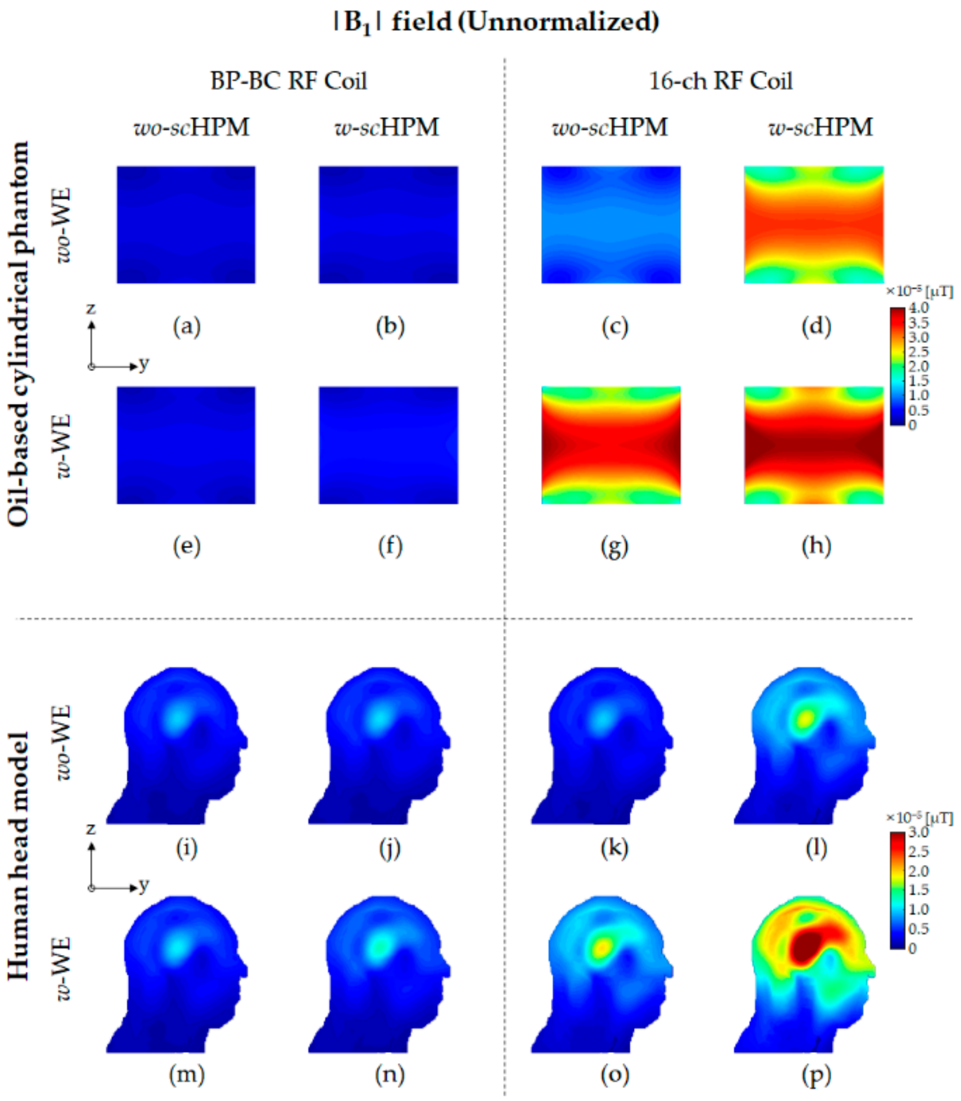

Figure S13. Unnormalized $|B_1|$ field distribution (*y*–*z* plane) in the oil-based cylindrical phantom (**a**–**h**) and human head model (**i**–**p**): (**a**,**i**) BP-BC RF coil – *wo-sc*HPM – *wo*-MCWE; (**b**,**j**) BP-BC RF coil – *w-sc*HPM – *wo*-MCWE; (**c**,**k**) 16-ch RF coil – *wo-sc*HPM – *wo*-BCWE; (**d**,**j**) 16-ch RF coil – *w-sc*HPM – *wo*-BCWE; (**e**,**m**) BP-BC RF coil – *wo-sc*HPM – *w*-MCWE; (**f**,**l**) BP-BC RF coil – *w-sc*HPM – *w*-MCWE; (**g**,**o**) 16-ch RF coil – *wo-sc*HPM – *w*-BCWE; (**h**,**p**) 16-ch RF coil – *w-sc*HPM – *w*-BCWE.
